# Cancer stem cells in hepatocellular carcinoma: therapy resistance and emerging treatments

**DOI:** 10.3389/fimmu.2025.1704868

**Published:** 2025-12-08

**Authors:** Dipesh Kumar Yadav, Rajesh Kumar Yadav, Alina Singh, Yi Huang, Dandan Bao, Zhangwei Yang, Hanzhang Huang, Yin Jiang, Pengwei Wang, Sisi Lin, Yongfei Hua, Yiren Hu

**Affiliations:** 1 Department of General Surgery, The Third Clinical Institute Affiliated to Wenzhou Medical University, Wenzhou People’s Hospital, Wenzhou, China; 2 College of Pharmacy, University of Louisiana at Monroe, Monroe, LA, United States; 3 Department of Surgery, Parkland Medicare and Research Center, Janakpur, Nepal; 4 Department of Hepatobiliary and Pancreatic Surgery, Ningbo Medical Center Lihuili Hospital, Ningbo University, Ningbo, Zhejiang, China

**Keywords:** cancer stem cells, liver cancer stem cells, hepatocellular carcinoma, liver cancer treatment resistance, immunotherapy

## Abstract

Hepatocellular carcinoma (HCC) imposes a significant global cancer mortality burden, with conventional therapies (surgery, ablation, chemotherapy, radiotherapy) and newer modalities (targeted agents, immune checkpoint inhibitors) limited by therapeutic resistance. Notably, liver cancer stem cells (Liver-CSCs)—defined by their self-renewal and unlimited proliferative capacity—drive tumor initiation, metastasis, heterogeneity, and therapy resistance. This review synthesizes current knowledge on Liver-CSCs, focusing on their distinctive features, supporting microenvironments, signaling pathways, and therapy resistance mechanisms. We also examine novel therapeutic strategies targeting these cells. Clinically, we evaluate recent research, identify knowledge gaps, and suggest potential directions for advancing HCC therapies. Finally, we discuss how these insights may inform development of more effective treatments to improve clinical HCC management. Understanding Liver-CSC biology and treatment resistance mechanisms will enable better-tailored therapies to overcome these challenges and enhance patient outcomes.

## 1 Introduction

Hepatocellular carcinoma (HCC), the most prevalent form of primary liver cancer, accounts for 75%-85% of liver cancer cases globally. As the sixth most common cancer worldwide, HCC poses a significant public health burden, with 865,269 new cases and 757,948 deaths reported in 2022 (1, 2).

The etiopathogenesis of HCC is multifactorial, including viral, metabolic, environmental, and genetic drivers. While chronic hepatitis B virus (HBV) and hepatitis C virus (HCV) infections dominate globally, accounting for 21%-55% of cases, with HBV endemicity driving over half of HCC diagnoses in East Asia and sub-Saharan Africa (2, 3). Emerging drivers such as metabolic dysfunction-associated steatotic liver disease (MASLD) and alcohol-associated hepatitis are reshaping HCC epidemiology in Western populations (4, 5). Environmental co-factors like dietary aflatoxin B1 synergize with HBV infection to amplify mutagenesis in sub-Saharan Africa and Southeast Asia (6). Genetic predisposition further modulates risk through tumor suppressor mutations (TP53), oncogenic pathway alterations (CTNNB1/β-catenin signaling), and germline variants in lipid metabolism (PNPLA3, TM6SF2) and lysosomal enzymes (CTSA) (7–9). Adeno-associated virus type 2 (AAV2) integration has also been implicated in HBV/HCV-negative HCC cases, suggesting a cooperative role in hepatocarcinogenesis (10). Critically, the cirrhotic microenvironment—marked by chronic inflammation, oxidative stress, and dysregulated repair—provides a permissive niche for malignant transformation (11).

Despite decades of therapeutic innovation, HCC management remains fraught with challenges. Current treatment modalities—including surgical intervention, ablation, chemotherapy, targeted therapies, and immunotherapies—are frequently compromised by high recurrence rates and intrinsic or acquired therapeutic resistance (12–14). Curative approaches such as surgical resection or liver transplantation are applicable to fewer than 30% of patients, while systemic therapies—encompassing multikinase inhibitors (e.g., sorafenib, lenvatinib) and immunotherapy (e.g., atezolizumab, nivolumab)—yield only transient clinical responses in 20%–30% of cases (15–18). Notably, five-year recurrence rates following curative resection reach as high as 50%–70% (19), and the emergence of therapy-resistant metastatic foci underscores the inadequacy of conventional strategies that target bulk tumor populations while overlooking reservoirs of treatment-evading cellular subclones.

The discovery of cancer stem cells (CSCs) in different cancers including Liver-CSCs in HCC, has provided transformative insights into tumor biology, particularly in understanding therapeutic resistance, recurrence dynamics, and metastatic dissemination (20–24). The CSC paradigm postulates that a hierarchically distinct cellular subset within the tumor microenvironment (TME) exhibits cardinal stemness properties—self-renewal, pluripotency, and asymmetric division—that orchestrate tumorigenesis, clonal evolution, and post-therapeutic relapse (20, 25, 26). Seminal work by Hermann et al. demonstrated the unparalleled tumor-initiating capacity of CD133^+^ CSCs: xenotransplantation of as few as 1,000 CD133^+^ cells sufficed for tumor engraftment in immunocompromised murine models, whereas 1 × 10^6^ CD133^−^ counterparts failed to propagate neoplasia (27). These findings have been robustly validated in independent studies employing analogous xenotransplantation methodologies (28, 29). In essence, such evidence posits Liver-CSC-directed therapeutic eradication as a pivotal strategy to achieve durable oncologic control in HCC.

This review provides a comprehensive analysis of Liver-CSCs, exploring their defining characteristics, supportive microenvironment, key signaling pathways, mechanisms of resistance to current therapies, and emerging therapeutic approaches. By evaluating the latest research, identifying critical knowledge gaps, and proposing future directions, this review aims to inform the development of more effective treatments for HCC.

## 2 From stem cell to Liver-CSC: a historical overview

**Figure 1:**
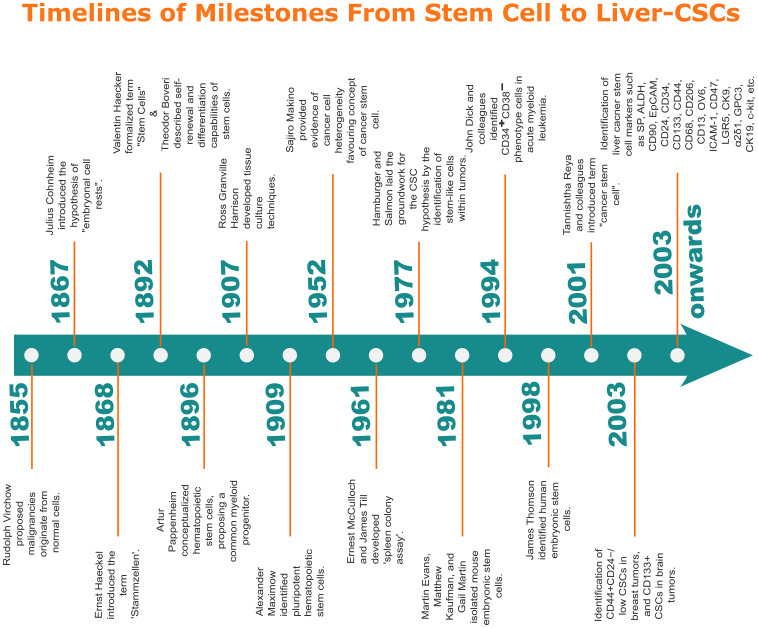
Key milestones in stem cell biology and liver-CSC discovery. Historical progression from foundational stem cell concepts to identification of Liver-CSC markers.

The conceptual and experimental evolution of stem cell biology, culminating in the CSC hypothesis, represents one of the most transformative narratives in modern oncology (**Figure 1**), beginning with Rudolph Virchow’s 1855 proposition that malignancies originate from normal cells—a cornerstone of cellular pathology that redefined cancer biology (30). Building on this framework, Julius Cohnheim postulated in 1867 that dormant embryonic cells (“embryonal rests”) could serve as latent tumor progenitors (31). Concurrently, Ernst Haeckel coined the term Stammzellen (stem cells) in 1868 to describe ancestral cells governing developmental hierarchies (32). By 1892, Valentin Haecker formalized “stem cell” terminology in embryological studies (33), while Theodor Boveri that same year detailed stem cell self-renewal and differentiation capacities, linking cellular hierarchy to tumorigenesis (33). In 1896, Artur Pappenheim conceptualized hematopoietic stem cells, proposing a common myeloid progenitor (34).

Experimental validation accelerated when Ross Granville Harrison developed tissue culture techniques in 1907, enabling stem cell isolation (35). Alexander Maximow’s 1909 identification of pluripotent hematopoietic stem cells (36). Further, in the 1950s, Sajiro Makino demonstrated cancer cell heterogeneity through serial murine ascites tumor transplantation, revealing subpopulations with distinct tumorigenic potential (23, 37). The 1960s witnessed Ernest McCulloch and James Till’s spleen colony assay, which established hematopoietic stem cell self-renewal (38). This groundwork culminated in 1977 when Hamburger and Salmon identified clonogenic, stem-like tumor cells (39).

The 1980s ushered in a new era of stem cell research. Martin Evans, Matthew Kaufman, and Gail Martin isolated mouse embryonic stem cells (ESCs) (40). Critical to CSC theory, John Dick’s team (1994) identified CD34^+^CD38^−^ leukemia-initiating cells in acute myeloid leukemia (AML) (25). This era was then eclipsed by James Thomson’s 1998 derivation of human ESCs (41) Tannishtha Reya formally unified these concepts under the term “cancer stem cell” (2001) (42), catalyzing landmark discoveries including Al-Hajj et al.’s 2003 isolation of CD44^+^CD24^−^/low breast CSCs and Singh et al.’s 2003 identification of CD133^+^ brain CSCs (43). These discoveries catalyzed further research, leading to the identification of CSCs in various cancers, including Liver-CSCs in HCC (44, 45).

Early discoveries in Liver-CSCs began in 2007 with the identification of CD133 as a marker for these cells. CD133^+^ cells were linked to high tumorigenic potential and poor prognosis in HCC (46). Subsequent studies unveiled a hierarchy of Liver-CSC markers, each revealing unique functional roles: CD90 (2008) linked to vascular invasion and metastasis through integrin-mediated adhesion (47); epithelial cell adhesion molecule (EpCAM) in 2009, associated with wingless-type (Wnt)/β-catenin-driven self-renewal (48); CD13 (2010) implicated in detoxifying oxidative stress to promote survival (49); and CD24 (2011) correlated with signal transducer and activator of transcription 3 (STAT3)-mediated metastatic plasticity (50). Since then, numerous studies have identified different unique Liver-CSCs markers and their associated features, which are explored in the subsequent sections of this review. These cells have become a central focus in HCC research due to their self-renewal (stemness), the capacity to adapt and multi-lineage differentiation potential (plasticity), and aggressive behavior—traits that are not only defining features but also crucial factors influencing the complex biology of HCC (20). The current understanding of Liver-CSCs is rapidly evolving, with recent research shedding new light on their molecular and cellular mechanisms that govern these cells, as well as their role in HCC.

## 3 Markers of Liver-CSCs

Liver-CSCs are characterized by a unique set of surface and intracellular markers that play a critical role in their identification and functional characterization (**Table 1**). Surface markers such as EpCAM (48, 51–54), cluster of differentiation 13 (CD13) (49). CD24 (50), CD34 (55, 56, 93), CD44 (44, 57, 58), CD47 (59–61), intercellular adhesion molecule-1 (ICAM-1, CD54) (62, 63), Thy-1 cell surface antigen (CD90) (64–66, 94), c-kit (CD117) (68), CD133 (prominin-1) (69, 70), CD206 (71), the oval cell marker (OV6) (72, 73, 95), glypican-3 (GPC3) (74, 96, 97), delta-like 1 homolog (DLK1) (75, 76, 98), alpha-2-delta-1 subunit of voltage-gated calcium channels (α2δ1) (77, 99), granulin-epithelin precursor (GEP) (78, 79), and leucine-rich repeat-containing G-protein coupled receptor 5 (LGR5) (80, 81, 100), along with intracellular markers like nestin (82), aldehyde dehydrogenase 1 (ALDH1) (83, 84), cytokeratin 19 (CK19,K19) (85, 101), K7 (86, 102), sal-like protein 4 (SALL4) (87, 88), maelstrom (MAEL) (89), DEAD box helicase 56 (DDX56) (90), brain-expressed X-linked protein 1 (BEX1) (91), and cripto-1 (92), are essential for defining the stem-like properties of Liver-CSCs and hold significant promise as therapeutic targets. Furthermore, core transcription factors—including octamer-binding transcription factor 4 (OCT4), nanog homeobox (NANOG), and sex-determining region Y box 2 (SOX2)—form a regulatory network essential for self-renewal, while krüppel-like factor 4 (KLF4) and cellular myelocytomatosis (c-MYC) enhance proliferation and survival. Together, they are critical functional markers of the tumor-initiating potential in Liver-CSCs (28).

**Table 1:** Table showing surface and intracellular markers identified in liver cancer stem cells (Liver-CSCs).

Markers	Functions in liver-CSCs	Ref.
Surface Markers		
Epithelial cell adhesion molecule (EpCAM)	Promotes stemness, tumorigenicity, proliferation, sphere formation, metastasis, angiogenesis, invasiveness, epithelial–mesenchymal transition (EMT), and chemo-/radioresistance.	(48, 51–54)
CD13	Maintains stemness and tumorigenicity, drives proliferation, induces cell-cycle arrest, and confers chemo-/radioresistance.	(49)
CD24	Sustains stemness and tumorigenicity, enhances proliferation, promotes chemoresistance, and facilitates metastasis.	(50)
CD34	Supports tumorigenicity, lineage plasticity, and angiogenesis.	(55, 56)
CD44	Maintains stemness and induces EMT.	(57, 58)
CD47	Maintains stemness and tumorigenicity, enables immune evasion, confers chemoresistance, and drives metastasis.	(59–61)
Intercellular adhesion molecule-1 (ICAM-1, CD54)	Sustains stemness and tumorigenicity, promotes sphere formation, and enhances metastasis.	(62, 63)
Thy-1 cell surface antigen (CD90)	Maintains stemness and tumorigenicity, stimulates proliferation, supports sphere formation, confers chemoresistance, and promotes metastasis.	(62, 64–67)
c-kit (CD117)	Maintains stemness, promotes angiogenesis, and induces EMT.	(68)
CD133 (Prominin-1)	Maintains stemness and tumorigenicity, drives proliferation, supports angiogenesis, facilitates metastasis and invasiveness, and confers chemo-/radioresistance.	(69, 70)
CD206	Maintains stemness and tumorigenicity, stimulates proliferation, and confers chemoresistance.	(71)
Oval cell marker (OV6)	Maintains stemness and tumorigenicity, confers chemoresistance, and promotes invasiveness and metastasis.	(72, 73)
Glypican-3 (GPC3)	Enhances tumorigenicity, inhibits apoptosis, and promotes invasiveness and metastasis.	(74)
Delta-like 1 homolog (DLK1)	Maintains stemness and tumorigenicity, supports sphere formation, and confers chemoresistance.	(75, 76)
Alpha-2-delta-1 subunit of voltage-gated calcium channels (α2δ1)	Sustains stemness and tumorigenicity.	(77)
Granulin-epithelin precursor (GEP)	Maintains stemness, supports sphere formation, and confers chemoresistance.	(78, 79)
Leucine-rich repeat-containing G-protein coupled receptor 5 (LGR5)	Maintains stemness and tumorigenicity, supports sphere formation, confers chemoresistance, and promotes invasiveness and metastasis.	(76, 80, 81)
Intercellular markers		
Nestin	Maintains stemness and tumorigenicity.	(82)
Aldehyde dehydrogenase (ALDH)	Sustains tumorigenicity, stimulates proliferation, and confers chemo-/radioresistance.	(83, 84)
Cytokeratin 19 or keratin 19 (CK19 or K19)	Enhances tumorigenicity and proliferation, confers chemoresistance, promotes invasiveness and metastasis, and induces EMT.	(76, 85)
Cytokeratin 7 (K7)	Promotes invasiveness and confers chemoresistance.	(86)
Sal-like protein 4 (SALL4)	Maintains stemness and tumorigenicity, stimulates proliferation, and confers chemoresistance.	(87, 88)
Maelstrom (MAEL)	Maintains stemness and tumorigenicity, stimulates proliferation, confers chemoresistance, promotes invasiveness and metastasis, and induces EMT.	(89)
DEAD box helicase 56 (DDX56)	Maintains stemness and tumorigenicity, stimulates proliferation, promotes invasiveness, supports sphere formation, induces EMT, and drives metastasis	(90)
Brain-expressed X-linked protein 1 (BEX1)	Maintains stemness and tumorigenicity.	(91)
Cripto-1	Maintains stemness and tumorigenicity, stimulates proliferation, promotes invasiveness, confers chemoresistance, and drives metastasis.	(92)

The significant heterogeneity of Liver-CSCs necessitates the use of a comprehensive panel of markers for accurate identification, as reliance on a single marker often fails to capture their full functional characteristics. For instance, while ALDH1 alone is unreliable (103), the CD133^+^ALDH^+^ population exhibits heightened tumorigenicity (83). Similarly, CD13^+^CD133^+^ co-expression markedly increases tumorigenic potential over single-positive cells (104). Critically, multi-parameter profiling reveals distinct functional hierarchies: CD133^+^EpCAM^+^ Huh7 cells exhibit superior differentiation, colony/spheroid formation, drug resistance, and tumorigenicity (105); CD24^+^CD133^+^ subsets correlate with worse clinical outcomes, elevated tumorigenicity, and sorafenib resistance (106); and an EpCAM^+^CD24^+^CD44^+^ subclone drives potent oncogenic circuits (107). Concurrently, CD90^+^CD44^+^ cells display enhanced aggressiveness and metastatic potential (64), while CD13^+^CD90^−^ cells exhibit greater self-renewal capacity and radiotherapy resistance than CD13^−^CD90^+^ cells (49). On the whole, these findings underscore the imperative for multi-marker strategies to precisely characterize Liver-CSCs and advance targeted liver cancer therapeutics.

## 4 Predictive value of Liver-CSCs in clinical practice

Liver-CSCs are pivotal biomarkers in HCC, with substantial evidence linking their expression to aggressive clinicopathological features and treatment resistance.

Elevated CD90 expression in HCC cells directly correlates with disease progression (108). Li et al. demonstrated that high CD90 levels in HCC are strongly associated with HBV infection and advanced histological grading (109). In a post-surgical cohort of 31 HCC patients, Luo et al. observed high CD90 expression in 5 patients, 80% (4/5) of whom exhibited venous infiltration; 50% (2/4) of these cases subsequently developed recurrence. By contrast, recurrence occurred in only 19% (5/26) of patients with low CD90 expression (67).

EpCAM-positive Liver-CSCs—whether detected as circulating tumor cells (CTCs) or within the TME—consistently associate with unfavorable prognosis, including reduced recurrence-free survival (RFS) and overall survival (OS) after curative resection (110–115). In a pilot study of 25 liver transplant patients, the presence of EpCAM^+^/CD90^+^ CTCs was significantly associated with HCC recurrence (116). Similarly, Kumagai et al. demonstrated that Liver-CSC marker expression correlates with tumor size and differentiation status. CD56 was predominantly expressed in larger, poorly differentiated tumors, while EpCAM was enriched in moderately differentiated HCC. Strikingly, well-differentiated tumors showed no detectable expression of these markers, suggesting that Liver-CSC marker acquisition occurs during tumor evolution rather than at initiation. This dynamic shift implies that Liver-CSC phenotypes emerge as tumors progress, reflecting a transition toward more aggressive biological behavior (117).

CK19^+^ Liver-CSCs, known to drive epithelial-mesenchymal transition (EMT) via transforming growth factor-β/small mothers against decapentaplegic (TGF-β/SMAD) signaling, associate with aggressive phenotypes including microvascular invasion, elevated alpha-fetoprotein (AFP) levels, and poor differentiation (118–122). A network meta-analysis further established CK19’s prognostic dominance: co-expression with EpCAM predicted the lowest OS (surface under the cumulative ranking curve [SUCRA]: 78.65%), while CK19 alone emerged as the strongest predictor of RFS (SUCRA: 98.93%) and disease-free survival (DFS) (SUCRA: 84.95%). Additionally, CD56, CK19, and CD133 were independently associated with poor differentiation, vascular invasion, and metastasis, respectively, underscoring their roles in tumor progression (85).

Sorafenib resistance strongly correlates with Liver-CSC biomarkers. Specifically, the overexpression of CD133 and CD90 associates with diminished sorafenib response, reduced OS, and therapeutic failure in sorafenib-treated advanced HCC (123, 124). Consequently, these markers may serve as biomarkers for sorafenib resistance. Further studies demonstrate that markers such as CD133, EpCAM, and CK19 are overexpressed in HCC patients following transarterial chemoembolization (TACE) treatment, associating these elevated levels with poor prognosis, recurrence, and treatment resistance (125–127). Tseeleesuren et al. specifically linked high CD133 expression to reduced post-TACE OS, tumor multiplicity, vascular invasion, and cirrhosis, whereas EpCAM overexpression—while associated with age, tumor burden, and viral status—showed no OS correlation (128).

Numerous studies have explored the clinical predictive value of CSCs in predicting radiotherapy responses. A systematic review identified CSC markers (CD133, CD44, ALDH1, LGR5) linked to radioresistance and poor prognosis in rectal cancer (129). Given similar cancer biology, these markers likely also predict radiotherapy response in HCC. Supporting this, CD133^+^ Liver-CSCs show greater resistance to radiation-induced apoptosis and enhanced tumor-proliferation capacity after radiation compared to CD133^-^ cells (130, 131).

Based on the discussed findings, predictive value of Liver-CSCs in clinical practice is substantial, with their expression patterns not only correlating with poor prognosis but also influencing treatment responses.

## 5 Platforms and technologies in the study of Liver-CSCs

Harnessing the power of modern biomedical research, the study of Liver-CSCs has entered a transformative era. Innovative platforms and technologies have unveiled critical insights into the pathways and processes that define the behavior of Liver-CSCs. Below, we explore key methodologies pivotal to Liver-CSC research, many of which are applicable to diverse cell populations.

Initial isolation approaches, such as density gradient centrifugation relying on biophysical properties like cell size and density (132, 133), are simple and induce minimal cellular damage but suffer from low specificity for CSCs (132). These have been largely superseded by antibody-based techniques—fluorescence-activated cell sorting (FACS) and magnetic-activated cell sorting (MACS)—which utilize surface markers for selective cell separation (134–136). MACS offers simplicity, sterility, and induces minimal cellular stress, but is generally restricted to single-marker isolation; conversely, FACS enables high-specificity, multi-marker sorting but is costly, imposes significant cell stress, requires stringent sterile conditions, and demands substantial cell inputs—a critical limitation given the scarcity of Liver-CSCs (137–139).

Functional characterization techniques include the side-population (SP) assay, which identifies Liver-CSCs via Hoechst 33342 dye efflux mediated by adenosine triphosphate (ATP)-binding cassette (ABC) transporters (e.g., ABCG2). This method isolates cells with chemoresistance and tumor-initiating capacity, which are linked to HCC recurrence and poor prognosis (140, 141). Similarly, the Aldefluor assay, which detects high ALDH activity to identify Liver-CSCs exhibiting enhanced self-renewal and invasiveness (142). While the SP assay is straightforward, its utility is constrained by dye toxicity and the heterogeneity of the isolated population, as the SP phenotype lacks universality and is susceptible to technical variability (143). Likewise, although ALDH activity is a strong functional indicator, it lacks universal specificity across all Liver-CSCs subtypes, as high ALDH activity is not exclusive to CSCs and may reflect contributions from multiple ALDH isoforms or activity in non-CSC populations. Additionally, the assay is susceptible to experimental variation and lacks standardized protocols (142).

Newer platforms circumvent marker dependence: microfluidics enables label-free isolation of cells using inherent biophysical properties such as deformability and adhesion, offering high-throughput processing but requiring specialized equipment and often yielding lower purity than antibody-based methods (144, 145). In parallel, intravital microscopy provides real-time, high-resolution visualization of cellular events within their native microenvironment, yielding crucial insights into their behavior and interactions. Its limitations, however, include a small field of view, limited penetration depth, phototoxicity, and clinical translation barriers due to fluorescent labeling requirements (146–148).

Molecular profiling utilizes single-cell RNA sequencing (scRNA-seq) to characterize transcriptional heterogeneity and rare subpopulations within their immune microenvironment, though it loses spatial context and requires advanced bioinformatics (149, 150). Complementarily, spatial transcriptomics maps Liver-CSC niche topography and localized gene expression but currently offers lower resolution and greater technical complexity (151, 152). Functional genomics employs clustered regularly interspaced short palindromic repeats (CRISPR)/CRISPR-associated protein 9 (Cas) screening for genome-wide identification of essential self-renewal regulators and vulnerabilities, though challenges include off-target effects and delivery efficiency (153–155). Meanwhile, proteomic and metabolomic profiling reveal druggable pathways and metabolic dependencies in Liver-CSCs, despite limitations in analyzing low-abundance proteins and metabolite instability (156–158).

Moving from analytical techniques to disease modeling, a major hurdle in Liver-CSC research is the lack of predictive models that truly capture tumor heterogeneity, reliably test drug response, and accelerate therapy discovery. While foundational, traditional 2D cultures and genetically engineered mouse models fail to mirror the intricate 3D architecture and metabolic functions of human liver tumors (159, 160). This has propelled the field toward more physiologically relevant systems.

Among advanced disease models, patient-derived xenografts (PDXs) marked a significant step forward. By transplanting patient tumors into mice, PDXs maintain the original cancer’s genetic and structural complexity *in vivo*, offering valuable insights for personalized medicine. However, their widespread use is limited by low engraftment success, high costs, long timelines, and the replacement of human stroma with mouse cells, which distorts the tumor microenvironment (160–162). These constraints make PDXs impractical for large-scale drug screening.

Consequently, patient-derived organoids (PDOs) have emerged as a flexible and scalable alternative. These 3D structures, grown directly from patient biopsies, preserve the diversity and plasticity of Liver-CSCs, making them powerful tools for studying therapy resistance (160, 163). A key focus is their potential to unravel the specific biology of Liver-CSCs and reveal new therapeutic targets (164). Despite this promise, current organoid models are often too simplistic, typically lacking the critical immune and stromal components of the native niche (160, 161). While genetically engineered and complex multi-cellular organoids are being developed to bridge this gap, they still struggle to fully replicate the mutational landscape of advanced tumors or maintain long-term stability (163, 165).

These advanced models form the foundation for high-throughput screening (HTS) platforms to identify Liver-CSC-specific drugs (166, 167). Yet, HTS in simplified organoid cultures can produce misleading results, as it cannot fully mimic the complex cell-cell interactions of a real tumor (168, 169). Computational approaches offer a complementary path, using machine learning to analyze large datasets, predict drug vulnerabilities, and design optimal combination therapies. However, their accuracy is constrained by data quality and the challenge of modeling the dynamic nature of cellular plasticity (170).

In concert, these synergistic methodologies are crucial for deciphering fundamental Liver-CSC pathobiology and translating mechanistic insights into targeted therapies. By integrating these complementary technologies, researchers can overcome their individual limitations in resolution, spatial context, and analytical depth, creating a more powerful and holistic research toolkit.

## 6 Origins of Liver-CSCs

**Figure 2:**
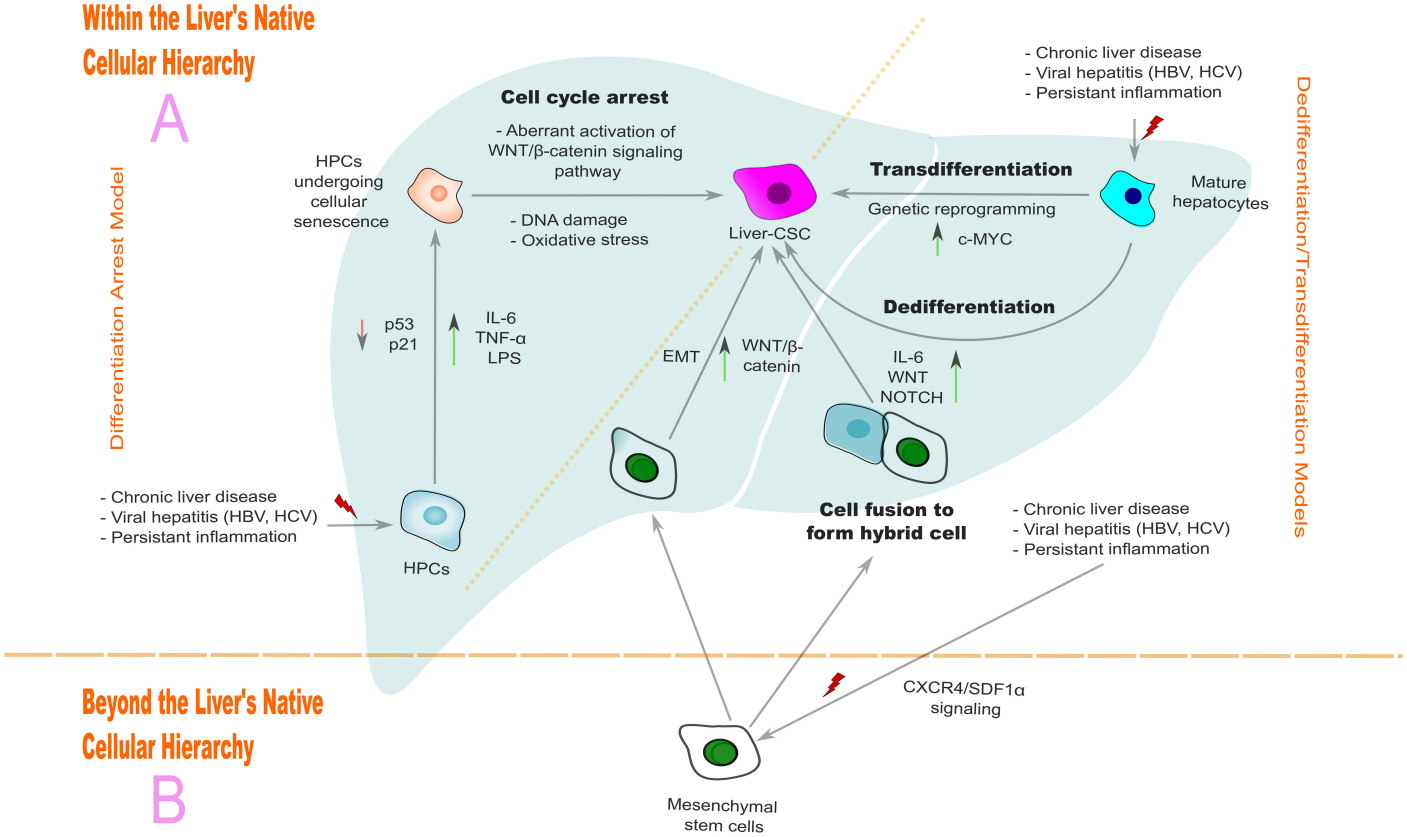
Proposed origins of liver-CSCs: intrahepatic and extrahepatic pathways. **(A)** Intrahepatic derivation: Hepatic progenitor cells (HPCs) undergo senescence-mediated differentiation arrest to generate Liver-CSCs, while mature hepatocytes acquire stem-like properties through dedifferentiation or transdifferentiation. **(B)** Extrahepatic derivation: Mesenchymal stem cells migrate to sites of hepatic injury, fuse with resident cells, and form hybrid Liver-CSCs. Chronic liver disease, HBV/HCV infections, and sustained inflammation initiate these pathogenic transformations.

Understanding the origins of Liver-CSCs is crucial for unraveling the complexities of HCC. The origins of Liver-CSCs are multifaceted, involving various cellular sources and intricate molecular mechanisms. Numerous theories have been proposed to explain their origins (**Figure 2**). However, these theories often intersect and share some common pathways, making it challenging to distinctly categorize them. This section explores the differentiation arrest, dedifferentiation, and transdifferentiation models within the liver’s native cellular hierarchy, as well as alternative theory involving extrahepatic sources.

### 6.1 Differentiation arrest and dedifferentiation/transdifferentiation models: within the liver’s native cellular hierarchy

The liver’s remarkable regenerative capacity is a vital asset, ensuring its functionality and recovery from various injuries. This process is primarily driven by mature hepatocytes, which are adept at proliferating and differentiating to restore the liver’s mass and function following injuries such as chemical toxicity or surgical resection (171). Meanwhile, hepatic progenitor cells (HPCs), also known as oval cells or liver stem cells, typically reside in a quiescent state within specialized niches, such as the canal of Hering (172, 173). These cells serve as a reserve population that can differentiate into hepatocytes and bile duct cells (cholangiocytes), thereby playing a pivotal role in liver regeneration and repair (172). Under normal physiological conditions, HPCs remain dormant, maintaining equilibrium within the liver’s cellular hierarchy. However, in cases of severe hepatocyte damage—such as that caused by cirrhosis, fibrosis, steatosis, inflammation, or viral infection—HPCs can be activated and mobilized to participate in the regenerative process (171, 172). The activation of HPCs is marked by the upregulation of biliary/progenitor cell markers, such as SOX9, K19, hepatocyte nuclear factor 1 beta (HNF1β), forkhead box L1 (FOXL1), and osteopontin (OPN) (174).

Building on this foundation, the differentiation arrest model has been proposed to explain the origin of liver-CSCs. This model posits that HPCs undergo cellular senescence, leading to cell cycle arrest when exposed to stressors such as carcinogens, persistent inflammation, oxidative stress, or prolonged exposure to gut-originated lipopolysaccharide (LPS) (175–178). Liu et al. demonstrated that LPS is a significant driver of HPC plasticity. Long-term exposure to LPS can cause HPCs to transform into myofibroblasts and tumor cells, potentially initiating HCC and enhancing its tumorigenesis (176).

Once HPCs enter this senescent state, tumor suppressor pathways like p53 and p21 are activated (177, 179). While these pathways initially serve a protective role, they can become dysfunctional due to DNA damage, mutations and other genetic alterations, ultimately contributing to the development of a malignant phenotype (179). This transition is often associated with the aberrant activation of the Wnt/β-catenin signaling pathway, which is a key driver of stem cell-like properties such as self-renewal and proliferation (177, 180). Senescent cells release a complex known as the senescence-associated secretory phenotype (SASP), which includes cytokines such as interleukin-6 (IL-6), IL-8, and tumor necrosis factor-alpha (TNF-α). These cytokines influence the TME facilitate immune evasion, promote tumor growth, and trigger angiogenesis. Furthermore, SASP enhances tumor cell stemness, leading to the emergence of aggressive, therapy-resistant tumor clones (177, 181–183).

In contrast, lineage tracing studies have challenged this paradigm, suggesting that HCC may predominantly originate from mature hepatocytes rather than HPCs (184, 185). This introduces the concepts of dedifferentiation and transdifferentiation, where hepatocytes, under chronic liver pathologies, may undergo cellular senescence and may revert to a stem-like state or transform into other cell types. Genetic reprogramming events, such as p53 loss and dysregulation of Wnt/NOTCH signaling, drive this process, conferring CSC-like properties (82, 181, 186–188). For instance, Karagonlar et al. demonstrated that KLF4 can induce dedifferentiation, transforming EpCAM^−^/CD133^−^ non-CSCs into EpCAM^+^/CD133^+^ Liver-CSCs in the HuH7 HCC cell line by acting as a transcriptional activator of the EpCAM gene (189). Similarly, chromodomain helicase/ATPase DNA binding protein 1-like (CHD1L) modulates chromatin architecture at loci encoding estrogen-related receptor beta (ESRRB) and transcription factor 4 (TCF4), enhancing tumorigenicity and disease progression (186). He et al. further identified hepatic cancer progenitor cells (HcPCs) within preneoplastic foci of altered hepatocytes (FAH), marked by CD44, EpCAM, and SOX9 expression, and underscored the pivotal role of IL-6 signaling in HcPC-driven tumorigenesis (190). Collectively, these findings highlight hepatocyte plasticity as a key driver of HCC progression. While the susceptibility of specific hepatocyte subtypes to transformation remains debated, both diploid and polyploid hepatocytes exhibit neoplastic potential (191, 192). Notably, pericentral hepatocytes (zone 3) demonstrate heightened oncogenic propensity, likely due to constitutive Wnt/β-catenin activation in this niche, though this warrants further investigation (193).

Thus, the cellular origin of Liver-CSCs—whether from HPCs or hepatocytes—remains contentious. However, growing evidence also suggests that diverse hepatic cell types—through genetic and epigenetic alterations—may acquire Liver-CSC properties, with c-MYC activation serving as a critical driver of reprogramming and tumorigenesis (194).

### 6.2 Alternative theories: beyond the liver’s native cellular hierarchy

Alternative theories have expanded the understanding of Liver-CSCs’ origin beyond the liver’s native cellular hierarchy, which propose that Liver-CSCs may originate from extrahepatic sources by process of cell fusion (93, 195–197).

The cell fusion hypothesis posits that Liver-CSCs can arise from the fusion of hepatic cells with extrahepatic cells, such as bone marrow-derived cells, myeloid lineage cells, or mesenchymal stem cells (MSCs). These extrahepatic cells are recruited to the liver during chronic liver injury, inflammation, or malignancy, primarily through the C-X-C motif chemokine receptor 4/stromal-derived factor 1 alpha (CXCR4/SDF1α) axis. Once in the liver, these cells can fuse with hepatocytes, HPCs, or transdifferentiated cells, resulting in the formation of hybrid cells with unique genomic and functional properties (195, 196, 198–200). This concept is supported by research on induced pluripotent stem cells (iPSCs), which demonstrates that differentiated cells can be reprogrammed into pluripotent states capable of generating various cell types, including those with CSC-like properties (201, 202). Similarly, hybrid cells formed as a result of cell fusion events exhibit pluripotency, genomic instability, and oncogenic potential, thereby contributing to the development and progression of HCC (203). Although physiological cell fusion is rare (e.g., in syncytiotrophoblast formation or myogenesis), chronic liver injury fosters a fusogenic milieu marked by upregulation of syncytins 1/2, annexins, myomaker, and myomerger, which mediate pathological fusion events (197, 203–205). The resultant hybrids acquire stem-like traits through integration of parental genomes, epigenetic remodeling, and activation of protumorigenic pathways such as Wnt, mitogen-activated protein kinase (MAPK), cyclic adenosine 3’,5’-monophosphate/protein kinase A (cAMP/PKA), and jun N-terminal kinase (JNK) (197, 203). Zeng et al. identified CD34^+^ Liver-CSCs arise from fusion between hepatobiliary progenitors and CD34^+^ hematopoietic precursors (93). These hybrids display canonical Liver-CSC features—stem marker expression, enhanced tumorigenicity, invasiveness, and chemoresistance—underscoring their role in HCC progression (93, 197, 200).

Overall, the origin of Liver-CSCs is characterized by a complex interplay of factors involving both intrahepatic and extrahepatic cells. This multifaceted perspective opens avenues for exploring the fundamental mechanisms of HCC development, with the potential to uncover novel therapeutic targets.

## 7 The Liver-CSCs niche

**Figure 3:**
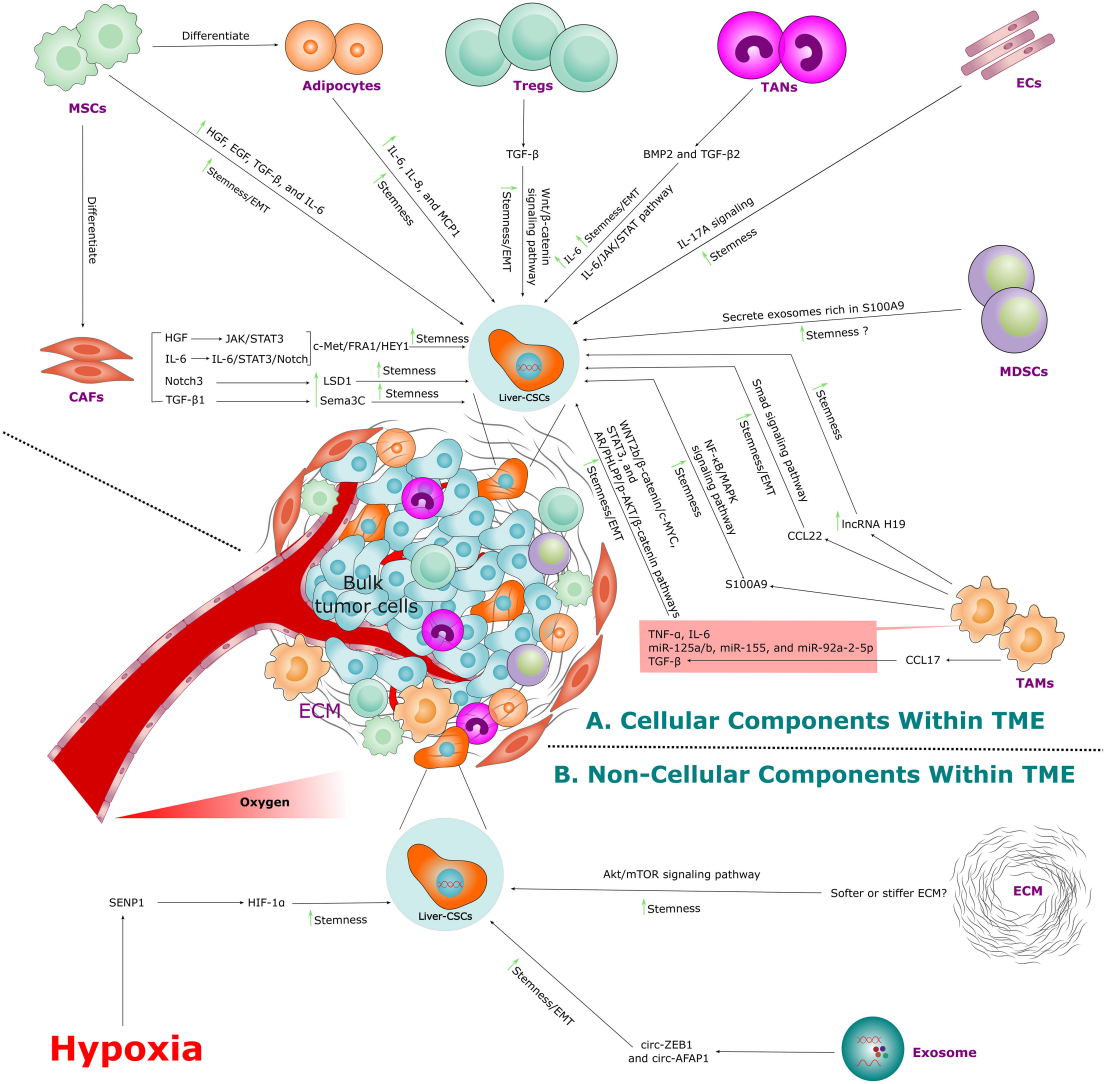
Liver-CSC niche architecture within the tumor microenvironment (TME). **(A)** Schematic of the cellular components that maintain Liver-CSC stemness, including cancer-associated fibroblasts (CAFs), mesenchymal stem cells (MSCs), adipocytes, regulatory T cells (Tregs), tumor-associated neutrophils (TANs), endothelial cells (ECs), myeloid-derived suppressor cells (MDSCs), and tumor-associated macrophages (TAMs), via paracrine signaling and direct interactions. **(B)** Schematic of the non-cellular components that reinforce Liver-CSC phenotypes, including hypoxia-mediated reprogramming, extracellular matrix (ECM) remodeling, and exosomal communication.

The Liver-CSC niche is a complex and dynamic microenvironment comprising cellular and non-cellular components that sustain Liver-CSC survival, self-renewal, and therapy resistance through structural support, molecular signaling, and protective mechanisms (**Figure 3**) (206–208). This niche critically shapes HCC progression by maintaining stemness and evading therapies. The following sections dissect these niche components, delineating their roles in maintaining Liver-CSC stemness and promoting tumor.

### 7.1 Cellular components of the Liver-CSCs niche

#### 7.1.1 Cancer-associated fibroblasts

Cancer-associated fibroblasts (CAFs) are phenotypically heterogeneous cell populations that play a central role in orchestrating the TME of HCC, impacting cancer cell behavior and progression (209). These cells arise from diverse cellular origins, including resident hepatic stellate cells (HSCs), MSCs, bone marrow-derived progenitors, and transdifferentiated local fibroblasts. However, the precise lineage hierarchy and contextual contributions of CAF precursors in HCC remain an area of active investigation (209–212).

CAFs are broadly classified into functionally distinct subtypes—such as myofibroblast-like (MyCAFs), inflammatory (iCAFs), and antigen-presenting (ApCAFs)—each defined by unique molecular signatures and tumor-modulatory roles (211, 213–215). MyCAFs, characterized by high expression of α-smooth muscle actin (α-SMA) and fibroblast activation protein alpha (FAP), drive desmoplastic stromal remodeling, which enhances tumor stiffness and restricts immune cell infiltration (211, 213, 216). In contrast, iCAFs secrete pro-inflammatory cytokines (e.g., IL-6, C-X-C motif chemokine ligand 12 (CXCL12)) to fuel tumor growth, while ApCAFs modulate adaptive immunity through antigen presentation, thereby shaping therapeutic responses in HCC (211, 214, 215). Notably, a recent multi-omics study integrating spatial transcriptomics, proteomics, and multiplexed imaging identified a novel CAF subset (F5-CAF) that directly promotes HCC cell proliferation and stemness via NOTCH and TGF-β signaling pathways (217).

CAFs are pivotal regulators of the Liver-CSC niche, where they sustain stemness, accelerate tumorigenesis, and confer therapy resistance via paracrine signaling networks (211, 217, 218). Preclinical 3D co-culture models combining murine LGR5^+^ Liver-CSCs with CAFs have demonstrated CAF-mediated amplification of Liver-CSC self-renewal and tumor-initiating capacity (219). Clinically, tripartite co-expression of CAF markers, lysine demethylase 1 (LSD1), and NOTCH3 in HCC specimens correlates with poor patient survival. Mechanistically, CAF-induced NOTCH3 activation drives LSD1-dependent epigenetic reprogramming of stemness-associated genes, thereby sustaining Liver-CSC aggressiveness (220).

CAFs deploy a repertoire of soluble mediators—including IL-6, hepatocyte growth factor (HGF), and TGF-β—to activate oncogenic pathways in Liver-CSCs (221, 222). For instance, CAF-derived IL-6 engages IL-6 receptors on CD24^+^ HCC cells, triggering STAT3 phosphorylation at Tyr705 via the IL-6/STAT3/NOTCH axis to amplify stem-like properties (221, 223). Similarly, CAF-derived HGF activates the cellular mesenchymal-epithelial transition factor (c-Met) receptor, inducing downstream signaling via FOS-related antigen 1 (FRA1) and hairy/enhancer-of-split-related transcription factor 1 (HEY1). This c-Met/FRA1/HEY1 axis governs Liver-CSC plasticity and survival in HCC (221, 224). Furthermore, TGF-β1 released by CAFs induces phosphorylation of SMAD2/3, thereby promoting stemness, EMT, and chemoresistance, which collectively reinforce the aggressive phenotype of Liver-CSCs (225, 226). Emerging evidence highlights that CAF-derived TGF-β1 stimulates semaphorin 3C (Sema3C) expression in HCC cells, establishing an autocrine signaling loop that perpetuates Liver-CSC stemness. Clinically, this Sema3C-driven mechanism correlates with increased tumor burden and dismal prognosis, underscoring its therapeutic relevance (222, 227).

Likewise, CAFs critically shape the vascular niche of Liver-CSCs by secreting pro-angiogenic factors such as vascular endothelial growth factor (VEGF) and stromal-derived factor 1 (SDF-1), which drive angiogenesis and vascular mimicry—processes linked to tumor aggressiveness and therapeutic resistance (228–230). Concurrently, CAF-mediated extracellular matrix (ECM) remodeling generates fibrotic barriers that physically shield Liver-CSCs from immune infiltration and biochemically impair drug penetration into tumor cores (231, 232).

These findings underscore the multifaceted role of CAFs in perpetuating Liver-CSC-driven HCC progression. Targeting CAF-Liver-CSC crosstalk constitutes a promising therapeutic strategy to disrupt tumor stemness, overcome immune evasion, and mitigate therapy resistance—a paradigm with profound implications for precision oncology.

#### 7.1.2 Mesenchymal stem cells

MSCs play a crucial role in HCC, exhibiting a context-dependent duality that modulates tumor progression. As previously discussed, MSCs inherently migrate to sites of hepatic injury and neoplastic foci, where they exhibit temporally divergent functions—suppressing tumorigenesis during early stages while paradoxically driving malignant progression in advanced HCC by expanding the Liver-CSC pool (199, 233–237). Through paracrine signaling, MSCs secrete a repertoire of cytokines and growth factors, including IL-6, HGF, epidermal growth factor (EGF), TGF-β, which activates oncogenic pathways such as Wnt/β-catenin, STAT3, and NOTCH. These pathways together enhance Liver-CSC self-renewal, stemness, and chemoresistance (235, 237, 238).

Concurrently, MSCs foster an immunosuppressive TME by expanding regulatory T cells (Tregs) and myeloid-derived suppressor cells (MDSCs) while suppressing cytotoxic T lymphocytes (CTLs) and natural killer (NK) cells, thereby facilitating immune evasion (237, 239). The plasticity of MSCs within the TME further amplifies their protumorigenic effects. Upon integration into hepatic tumor niches, MSCs undergo phenotypic transformation into CAFs and adipocytes, directly modulating Liver-CSC behavior and promoting EMT, angiogenesis, and metastatic dissemination (235, 237, 239).

Notably, the interplay between MSC-derived adipocytes and hepatocarcinogenesis is particularly salient in MASLD, where chronic inflammation and adipose accumulation elevate HCC risk. Preclinical studies in HCV-NS5A transgenic mice demonstrate that a cholesterol- and saturated fat-enriched diet (HCFD) accelerates Liver-CSC expansion and tumorigenesis. Mechanistically, HCFD activates the TLR4-NANOG and leptin receptor (OB-R)-pSTAT3 signaling axes, enriching twist-related protein 1 (TWIST1)-expressing Liver-CSC populations (240). Furthermore, adipocyte-secreted cytokines—including IL-6, IL-8, and monocyte chemoattractant protein 1 (MCP1)—expand the EpCAM^+^CD133^+^ HCC subset, which acquires migratory properties and sorafenib resistance via c-Met, STAT3, and extracellular signal-regulated kinase 1/2 (ERK1/2) pathway activation (241).

The dual role of MSCs in HCC underscores their potential as both mediators of disease progression and therapeutic targets.

#### 7.1.3 Tumor-associated macrophages

Tumor-associated macrophages (TAMs) are a critical component of the HCC microenvironment, where they intricately interact with Liver-CSCs to modulate stemness, tumor progression, sphere formation, EMT, and metastasis through diverse molecular interactions (242, 243). TAMs in HCC originate from two primary sources: Kupffer cells— resident tissue macrophages (RTMs), which adopt protumorigenic phenotypes under chronic inflammation, and bone marrow-derived monocytes that differentiate into TAMs upon migration to the liver in response to inflammatory or injury signals during liver cancer (242, 244). These cells exhibit functional plasticity, broadly categorized into M0, M1, or M2 states in response to microenvironmental cues (242, 244, 245).

M0 macrophages, enriched in HCC TME, serve as a baseline reservoir (non-activated) primed to respond to pathogenic or homeostatic signals, differentiate into M1 or M2 subtypes based on encountered stimuli (245, 246). Classically activated M1-like macrophages exhibit pro-inflammatory properties and shape the immune landscape of the TME. They are activated by interferon gamma (IFN-γ) or LPS, secrete pro-inflammatory cytokines (IL-1β, IL-1α, IL-6, IL-12, IL-18, IL-23, TNF-α) and chemokines (CXCL1,CXCL3, CXCL5, CXCL8,CXCL9, CXCL10, CCL4, CCL5, CCL8, CCL10, CCL11) that recruit CTLs and NK cells (244), yet paradoxically foster immune evasion through IL-1β-mediated programmed death-ligand 1 (PD-L1) upregulation on Liver-CSCs via interferon regulatory factor 1/nuclear factor kappa B (IRF1/NF-κB) signaling (247).

In contrast to M1-like macrophages, M2-like macrophages are alternatively activated exhibit anti-inflammatory and protumorigenic properties. Phenotypically, they are considered to be TAMs (242, 248). M2-like TAMs, polarized by cytokines such as IL-4, IL-10, IL-13, TGF-β, or prostaglandin E2 (PGE2), express surface markers (CD64, CD206, CD209, CD163) and secrete immunosuppressive mediators (IL-10, TGF-β, CCL17, CCL18, CCL22, CCL24). These factors drive type 2 helper T (Th2) cell polarization, angiogenesis, and fibrosis while also shielding Liver-CSCs from immune surveillance (244, 249). Additionally, IL-6, TNF-α, and TGF-β secreted by M2-like TAMs activate STAT3, NF-κB, and Wnt/β-catenin pathways in Liver-CSCs, thereby reinforcing stemness, EMT, and sorafenib resistance (243, 250, 251). TAM-derived S100 calcium-binding protein A9 (S100A9) further amplifies Liver-CSCs aggressiveness through NF-κB and MAPK signaling (252, 253). Notably, emerging studies highlight that TAMs-induced long non-coding RNA (lncRNA) H19 up-regulation promotes stemness and tumorigenesis in HCC cells via the miR-193b/MAPK1 pathway (254).

Notably, M0-like macrophages, though historically understudied, are enriched in HCC tissues compared to normal liver and are implicated in early tumor development, immune evasion, and correlate with poor patient survival (246). Liver-CSCs secrete CCL15, which polarizes M0 macrophages into SPP1^+^ TAMs—a hypoxic niche-associated subset regulated by hypoxia-inducible factor 1-alpha (HIF-1α). SPP1^+^ macrophages co-localize with Liver-CSCs and secrete vitronectin (VTN), activating the integrin αvβ5/AMP-activated protein kinase (AMPK)/Yes-associated protein 1 (YAP1)/SOX4 pathway to drive stemness and tumor progression. Their expression of matrix metalloproteinase-9 (MMP-9), MMP-12, and MMP-7 further contributes to immunotherapy resistance (255, 256).

Altogether, these findings underscore TAMs as important regulators of the Liver-CSC niche, with precision targeting of their dynamic crosstalk offering a transformative approach to overcoming HCC therapy resistance.

#### 7.1.4 Regulatory T cells

Tregs function as master suppressors of antitumor immunity within the Liver-CSCs niche, establishing an immune-privileged sanctuary that perpetuates HCC progression. Defined by their expression of FOXP3, CD25, and cytotoxic T-lymphocyte-associated protein 4 (CTLA-4), Tregs infiltrate the TME via chemokine gradients (e.g., CCL17, CCL22) secreted by Liver-CSCs and stromal cells (257, 258). Within this niche, they employ several key immunosuppressive mechanisms: (1) direct suppression of CTLs and NK cells through CTLA-4 and lymphocyte activation gene 3 (LAG-3) checkpoint signaling (259, 260) (2); secretion of IL-10, TGF-β, and IL-35 to polarize macrophages toward an M2-like phenotype and inhibit dendritic cell (DC) maturation (244, 258, 261, 262); and (3) metabolic sabotage via CD39/CD73-mediated adenosine production, which induces T-cell anergy through adora2A (A2A) receptor activation (263).

Beyond suppressing antitumor immunity, Tregs directly amplify Liver-CSC stemness. A recent study demonstrated that Tregs inhibit FOXP3 expression in HCC cells, destabilizing glycogen synthase kinase-3β (GSK-3β) and stabilizing β-catenin. This activates Wnt signaling, upregulating TIC markers (e.g., CD133, OCT3/4) and inducing EMT. This FOXP3/β-catenin axis drives a pronounced “stemness” phenotype, evidenced by increased CSC ratios, enhanced tumor sphere formation, and elevated tumorigenic capacity *in vivo* (264).

In conclusion, Tregs play a multifaceted role in the Liver-CSC niche, promoting immunosuppression while simultaneously enhancing the stemness and survival of Liver-CSCs through diverse molecular interactions and signaling pathways.

#### 7.1.5 Tumor-associated neutrophils

In HCC, tumor-associated neutrophils (TANs) have been classically defined by their dual roles: the anti-tumorigenic N1 phenotype and the protumorigenic N2 phenotype, which dynamically shift as the disease progresses (187, 188). Recent breakthroughs, however, have unveiled a far more complex landscape. Single-cell transcriptomic profiling has identified 11 distinct neutrophil clusters within HCC, each exhibiting unique molecular signatures and spatial localization—some enriched in peripheral blood, others in adjacent liver tissue, and subsets infiltrating tumor cores (265). Despite this heterogeneity, the N1/N2 paradigm remains foundational for understanding TAN interactions within the HCC microenvironment, a critical hub for tumor recurrence and therapy resistance.

TAN recruitment to the TME is orchestrated by tumor-derived chemokines (CXCL1, CXCL5, CXCL8) binding to CXCR2 on neutrophils, alongside granulocyte colony-stimulating factor (G-CSF), which amplifies their mobilization from bone marrow. These mechanisms prime TANs for context-dependent roles, ranging from tumor suppression in early HCC to fostering metastasis and immunosuppression in advanced disease (266, 267).

During early HCC, N1 TANs exert anti-tumor activity through the production of cytotoxic mediators, including reactive oxygen species (ROS), reactive nitrogen species (RNS), hydrogen peroxide (H_2_O_2_), and TNF-α, which directly induce oxidative stress, DNA damage, and apoptosis in cancer cells (268–270). Additionally, antibody-dependent cellular cytotoxicity (trogoptosis)—mediated by neutrophil Fcγ receptors binding to antibody-opsonized tumor cells—enhances their tumoricidal capacity during antibody-based therapies (271). Concurrently, N1 TANs amplify anti-tumor immunity by recruiting CD8^+^ T cells via chemokines such as CXCL10 and CCL3, facilitating antigen-specific tumor clearance (268, 272).

However, in advanced HCC, tumor-derived signals—including TGF-β and granulocyte-macrophage colony-stimulating factor (GM-CSF)—reprogram N1 TANs toward the N2 phenotype (273). These N2 TANs drive angiogenesis by secreting MMP-9. MMP-9 degrades the ECM, releasing sequestered VEGF to stimulate endothelial cell proliferation and neovascularization (274, 275). Concurrently, they establish an immunosuppressive microenvironment through arginase-1-mediated arginine depletion, which impairs T-cell function (270), and recruit tumor-promoting macrophages and Tregs, fostering tumor growth and sorafenib resistance (276). N2 TANs further promote metastasis by secreting oncostatin M (OSM) and HGF, which induce EMT in tumor cells (277). Crucially, N2 TANs engage in crosstalk with Liver-CSCs by releasing bone morphogenetic protein 2 (BMP2) and TGF-β2, which upregulate microRNA (miRNA)-301-3p expression in HCC cells. This miRNA suppresses limbic system-associated membrane protein (LSAMP) and cylindromatosislysine 63 deubiquitinase (CYLD), enhancing Liver-CSC stemness and tumor aggressiveness (278).

#### 7.1.6 Endothelial cells

The tumor vasculature is a cornerstone of the TME, dynamically shaped by endothelial cells (ECs) that originate from both pre-existing vasculature and bone marrow-derived endothelial progenitor cells (EPCs) (279, 280). Within the TME, ECs transform into tumor endothelial cells (TECs), acquiring pro-angiogenic and immunosuppressive properties distinct from normal ECs (281–283). TECs secrete angiogenic mediators such as VEGF, basic fibroblast growth factor (bFGF), and angiopoietin to drive abnormal vessel growth and enhance vascular permeability, while simultaneously downregulating adhesion molecules, including E-selectin, vascular cell adhesion molecule-1 (VCAM-1), and ICAM-1, to impair immune cell infiltration and promote immune evasion (283). Beyond fostering angiogenesis, TECs exhibit remarkable plasticity: they undergo endothelial-to-mesenchymal transition (EndoMT) to generate CAFs, which facilitate tumor invasion and therapy resistance (279, 284). Critically, TECs further drive immunosuppression by expressing PD-L1, which suppresses T-cell activity and enriches Tregs (279). Additionally, a distinct subset of CXCL12^+^ TECs has recently been identified that plays a pivotal role in sustaining immune suppression within the HCC microenvironment, thereby exacerbating immune evasion (285).

Bidirectional crosstalk between TECs and Liver-CSCs underpins the vascular niche’s role in tumor survival and therapy resistance. For example, lymphatic ECs promote Liver-CSC self-renewal and immune evasion via IL-17A signaling (286). Conversely, Liver-CSCs can transdifferentiate into TECs through ROS-dependent activation of the protein kinase B/inhibitor of κB kinase (AKT/IKK) pathway (287, 288). Liver-CSCs also enhance angiogenesis by releasing exosomes containing lncRNAH19, which upregulates VEGF receptor 1 (VEGFR1) in TECs, fostering metastasis, recurrence, and resistance to anti-angiogenic therapies (289).

In summary, ECs within the Liver-CSC niche are dynamic, multifunctional regulators of tumor progression. Through molecular crosstalk, phenotypic plasticity, and immunosuppression, they orchestrate tumor growth, immune evasion, and metastasis, highlighting their potential as therapeutic targets.

#### 7.1.7 Myeloid-derived suppressor cells

Myeloid-derived suppressor cells (MDSCs) are a heterogeneous population of immature myeloid cells that critically regulate immunosuppression in the TME. Originating from myeloid precursors, MDSCs expand pathologically in cancer, chronic inflammation, and autoimmune diseases (290–293). Beyond suppressing antitumor immunity, MDSCs drive cancer progression by promoting angiogenesis, enhancing tumor cell invasion and metastasis, and conferring resistance to chemotherapy (293–295). These cells are broadly classified into polymorphonuclear (PMN-MDSCs) and monocytic (M-MDSCs) subsets (293), distinguished by phenotypic and functional differences. In humans, PMN-MDSCs are characterized as CD11b^+^CD14^−^CD15^+^ or CD11b^+^CD14^−^CD66b^+^, while M-MDSCs are defined as CD11b^+^CD14^+^HLA-DR^−/low^CD15^−^, CD33^+^HLA-DR^−^Lin^−^, or CD14^+^HLA-DR^-/low^ (296). Additionally, a fibrocystic subset (F-MDSCs) has been described, contributing to the immunosuppressive network within the TME (297). These cells suppress antitumor immunity, promote angiogenesis, and confer therapy resistance through transcriptional dysregulation (interferon regulatory factor 8 (IRF8) downregulation, CCAAT/enhancer-binding protein beta (C/EBPβ) upregulation) and activation of NOTCH, adenosine A2B receptor, and NLR family pyrin domain-containing 3 (NLRP3) pathways (293, 298, 299).

In HCC, MDSCs exhibit elevated PD-L1 expression, driven by hypoxia and IL-6, which suppresses T-cell activity via programmed death 1 (PD-1) binding and expands Treg populations (300–302). Functionally, M-MDSCs upregulate arginase-1 (ARG1) to deplete arginine and secrete TGF-β and IL-10, thereby inhibiting both antigen-specific and nonspecific T-cell responses. In contrast, PMN-MDSCs generate high levels of ROS and RNS, primarily suppressing antigen-specific T-cell activity (303). M-MDSCs additionally impair NK cell cytotoxicity via NKp30 receptor interactions (304). Spatially, PMN-MDSCs localize to peripheral lymphoid tissues with modest suppressive activity, while M-MDSCs infiltrate tumors, undergo rapid differentiation into TAMs, and acquire enhanced immunosuppressive potency on a per-cell basis (68).

Hypoxia-driven CCL26 and VEGF derived from Liver-CSC-derived recruit MDSCs to the TME in HCC (305, 306), while tumor-secreted stem cell factor (SCF) and IL-6 expand MDSC populations and amplify immunosuppression (307, 308). Clinically, MDSC abundance correlates with advanced disease stages and poor patient survival (309). Mechanistically, MDSCs drive sorafenib resistance by upregulating fibroblast growth factor 1 (FGF1), which activates CAFs to sustain tumor growth (310).

MDSCs also play a crucial role in modulating CSC stemness across various malignancies. For instance, hypoxia-induced S100A9-rich exosomes enhance colorectal CSC self-renewal (311). In multiple myeloma, piwi-interacting RNA-823 (piRNA-823) expression in MDSCs promotes DNA methylation, sustaining CSC plasticity (312). In intrahepatic cholangiocarcinoma (IHCC), CAF-educated MDSCs upregulate 5-lipoxygenase (5-LO), producing leukotriene B4 to reinforce CSC traits (313). Similar mechanisms involving STAT3 and NOTCH pathways drive CSC stemness in ovarian, pancreatic, breast, and esophageal cancers (314–317). Although direct evidence in Liver-CSCs is limited, these findings suggest that MDSCs may also contribute to HCC through analogous crosstalk, positioning them as pivotal therapeutic targets.

#### 7.1.8 Hepatic stellate cells

HSCs, resident liver pericytes, are central to hepatic homeostasis, regulating retinoid storage and immune modulation. Upon liver injury, quiescent HSCs undergo activation, transitioning into myofibroblast-like cells that drive ECM deposition—a process critical for tissue repair but pathogenic in chronic settings, fostering fibrosis and cirrhosis (318–321). Recent findings highlights dynamic crosstalk between activated HSCs and Liver-CSCs. Activated HSCs exhibit phenotypic plasticity, with potential transdifferentiation into progenitor-like cells mediated by stemness-associated pathways (Hedgehog (Hh), Wnt) and markers (e.g., CD133), though their direct role in hepatocarcinogenesis remains under investigation (322–325).

Beyond fibrosis, HSCs contribute to immune evasion via PD-L1 expression, which induces T-cell apoptosis, and to chemoresistance through HGF secretion. Yu et al. demonstrated that HSC-derived HGF activates MET signaling in HCC cells, triggering EMT and enriching CSC-like properties, thereby reducing cisplatin sensitivity (326, 327). These findings underscore HSCs as multifunctional orchestrators of HCC progression, influencing tumor plasticity, immunosuppression, and therapeutic resistance.

### 7.2 Non-cellular components of the Liver-CSCs niche

#### 7.2.1 Extracellular matrix

The ECM, a dynamic network of collagens, laminins, fibronectin, proteoglycans, and glycosaminoglycans, serves as a structural and functional scaffold within the TME, organizing tumor cells—including Liver-CSCs—and regulating their proliferation, differentiation, and resistance to apoptosis (328–330). Cancer cells and stromal cells actively remodel the ECM through deposition, modification, and degradation, altering its composition, architecture, and biomechanical properties. These changes influence tumor progression by modulating cell migration, metastatic potential, and drug resistance (329–332). ECM stiffness, in particular, exhibits a complex relationship with Liver-CSC behavior. While metastatic HCC tissues often display increased stiffness correlated with poor prognosis (333–335), studies report conflicting effects: softer ECM may promote stemness in some contexts, whereas stiffer ECM enhances Liver-CSC traits in others (333, 336). For example, stiff matrices activate integrin β1-dependent AKT and mechanistic target of rapamycin (mTOR) signaling, reinforcing cancer stemness and reducing sorafenib-induced apoptosis (335, 336). Li et al. further demonstrated that stiff ECM directly amplifies Liver-CSC self-renewal compared to softer ECM (337). The ECM also acts as a physical barrier, shielding Liver-CSCs from cytotoxic therapies and immune cell infiltration (332).

Liver-CSCs dynamically remodel the ECM to adapt to microenvironmental demands. CD133^+^ subsets generate soft ECM microdomains through matrix degradation, fostering stemness maintenance, drug resistance, and metastasis (338). Conversely, α2δ1^+^ Liver-CSCs secrete lysyl oxidase (LOX) to stiffen the ECM, a process critical for acquiring and sustaining stem-like properties (99). Specific ECM components, such as laminin-3’s γ2 chain and collagen type 1 α1 (COL1A1), further promote stemness, offering potential therapeutic targets (339, 340). Beyond biochemical signaling, the ECM provides mechanical anchorage for cell division and metastatic colonization, underscoring its dual role as a biochemical and biomechanical regulator of tumor dissemination (332, 341).

#### 7.2.2 Hypoxia

Hypoxia is a critical non-cellular component of the Liver-CSC niche, driving stemness, therapy resistance, and immune evasion through the central role of HIFs (342–344). These heterodimeric proteins, composed of HIF-1/-2α and HIF-1β subunits (345, 346), execute distinct functions: HIF-1α redirects cellular metabolism toward glycolysis, while HIF-2α enhances expression of pluripotency factors to maintain the stem-like state (346, 347). This metabolic reprogramming upregulates key glycolytic enzymes—including hexokinase 2 (HK2), pyruvate kinase (PK), and lactate dehydrogenase A (LDHA)—and glucose transporters GLUT1 and GLUT3, resulting in increased glucose uptake and lactate secretion (348). The resultant acidification of the TME suppresses antitumor immunity and fosters an immunosuppressive milieu by recruiting immunosuppressive cells such as TANs, macrophages, and MSCs, while activating CAFs to promote therapy resistance, recurrence, and metastasis (349–353).

Moreover, hypoxia drives phenotypic plasticity through dedifferentiation of non-CSCs into stem-like states (“phenotype switching”) (344, 354–357). This reprogramming is mediated by hypoxia-induced upregulation of pluripotency-associated transcription factors (OCT4, NANOG, SOX2, KLF4, c-MYC) and miRNAs (e.g., miR-302), which stabilize HIF-1α to amplify its transcriptional activity. These molecular alterations enhance aggressiveness, invasiveness, and metastatic potential of CSCs like Liver-CSCs while reinforcing their stemness, survival, and resistance to therapy (344, 358–363). Central to this mechanism, sentrin-specific protease 1 (SENP1), a deSUMOylation enzyme, enhances hypoxia-induced Liver-CSC stemness by deSUMOylating HIF-1α. This action establishes a positive feedback loop that perpetuates stem-like characteristics (363).

A key consequence of hypoxia is vasculogenic mimicry, wherein tumor cells form endothelial-independent, blood vessel-like structures via epithelial-to-endothelial transition (EET) (364–366). Regulated by HIF-α, metastasis-associated lung adenocarcinoma transcript 1 (MALAT1), TWIST1, snail family transcriptional repressor 2 (Slug), phosphatidylinositol 3-kinase (PI3K), AKT, and PKA (364, 365, 367), vasculogenic mimicry generates mosaic vessels that facilitate the shedding of tumor cells into the circulation, thereby promoting metastasis (368).

In summary, hypoxia drives Liver-CSCs into a more aggressive and therapy-resistant state, making them a critical target in HCC treatment strategies.

#### 7.2.3 Extracellular vesicles

Extracellular vesicles (EVs), including exosomes and microvesicles, are nano-sized vesicles released by cells that carry a diverse array of molecular cargo reflective of their cellular origin (369). This cargo can offer a unique ‘signature’ reflecting tumor development, metastatic progression, and the metabolic status of the tumor cells (368, 370). Although the exact mechanisms of exosome packaging are not fully understood, it is evident that metastatic tumor cells possess a high capacity for packaging and secreting cargo, including proteins, RNA, DNA, and metabolites, within exosomes (368, 371). This ability to package and secrete a variety of biomolecules contributes to the role of exosomes in intercellular communication and their potential as biomarkers and therapeutic targets in cancer (368, 372). Notably, a recent study revealed that highly metastatic HCC cells release exosomes with reduced levels of transmembraneserine protease 2 (TMPRSS2) compared to less aggressive counterparts (373). Exosomes enriched in TMPRSS2 were found to inhibit packaging of the protumorigenic protein nidogen 1 (NID1) while suppressing proliferation and migration of immortalized liver cells (373).

Additionally, cancer-derived exosomes transport oncogenic transcription factors (e.g., NANOG, OCT4, Wnt proteins) that induce self-renewal and stem-like traits in recipient cells via genetic reprogramming (374, 375). Han et al. identified Liver-CSC-derived circular RNAs (circRNAs) such as circ-ZEB1 and circ-AFAP1 via immunomagnetic sorting and sequencing (376); these circRNAs promote stemness, tumor progression, and EMT, correlating with poor prognosis (376). Similarly, HCC EVs deliver miR-3129 to suppress thioredoxin-interacting protein (TXNIP), inhibiting apoptosis and stimulating proliferation (377). Parallel studies show EV-mediated delivery of miR-584-5p drives angiogenesis by inhibiting nuclear factor erythroid 2-related factor 2 (NRF2) through phosphoenolpyruvate carboxykinase 1 (PCK1) suppression in endothelial cells (378).

Critically, Huang et al. demonstrated that RAB27A-dependent exosome secretion from Liver-CSCs induces NANOG expression in differentiated liver cancer cells, conferring regorafenib resistance (379). NANOG depletion restores regorafenib sensitivity despite Liver-CSC exosome exposure (379), indicating that disrupting the RAB27A-exosome-NANOG axis targets a core Liver-CSC survival mechanism.

In brief, these findings demonstrate that EVs critically orchestrate the Liver-CSC niche through multifaceted mechanisms: delivering stemness-inducing factors, remodeling the TME, and conferring therapy resistance.

## 8 Factors regulating properties of Liver-CSCs

The maintenance and aggressiveness of Liver-CSCs are governed by a complex, multi-layered regulatory network, including signaling pathways, epigenetic mechanisms, and metabolic reprogramming. Understanding this hierarchical yet reciprocal network is paramount for developing effective strategies to target this resilient cell population.

### 8.1 Signaling pathways

Oncogenic signaling pathways form the regulatory foundation of the Liver-CSC state, engaging in extensive crosstalk to establish robust networks that enforce cellular aggressiveness.

The Wnt/β-catenin pathway serves as a cornerstone of Liver-CSC maintenance. Its aberrant activation through mutations in key regulators—including adenomatous polyposis coli (APC), axis inhibition protein (AXIN), and CTNNB1—drives HCC progression, therapy resistance, and disease recurrence (78, 380–383). The pathway further reinforces stemness through positive feedback loops; the Eph receptor B2 (EPHB2)-T cell factor 1 (TCF1) circuit creates a self-sustaining cycle that perpetuates the stem-like state (384). Notably, established Liver-CSC markers function within this pathway: EpCAM and Cripto-1 interface with core receptors (frizzled class receptor 7 (FZD7) and low-density lipoprotein receptor-related protein 6 (LRP6)) and transducer proteins (disheveled segment polarity protein 3 (DVL3)) to modulate stemness, proliferation, migration, invasion, and therapy resistance (78, 92, 385, 386). Furthermore, regulatory proteins including sirtuin-1 (SIRT1) contribute to Liver-CSC self-renewal by stabilizing β-catenin (387). Separately, protein tyrosine kinase 2 (PTK2) promotes HCC tumor growth and sustains Liver-CSC characteristics by augmenting the Wnt/β-catenin pathway. Specifically, PTK2 facilitates β-catenin nuclear translocation, thereby enhancing sorafenib resistance and reinforcing the Liver-CSC phenotype (388).

The Janus kinase (JAK)/STAT pathway, particularly STAT3, integrates inflammatory signals to drive Liver-CSC maintenance. STAT3 activation promotes stemness in EpCAM^+^/CD133^+^ Liver-CSCs through SOX4 upregulation, while in CD24^+^ cells, it drives tumorigenesis via NANOG expression (50, 389). This pathway additionally confers chemoresistance through glycochenodeoxycholic acid (GCDC)-mediated downregulation of negative regulators suppressor of cytokine signaling 2 (SOCS2) and protein tyrosine phosphatase non-receptor type 1 (PTPN1) (390). Furthermore, the IL-6/STAT3 axis maintains Liver-CSC populations through crosstalk with TGF-β signaling while promoting immune evasion via PD-L1 upregulation (391, 392).

The TGF-β signaling pathway, predominantly mediated through canonical SMAD-dependent signaling, exhibits a context-dependent duality in hepatocarcinogenesis. During early tumorigenesis, TGF-β signaling acts as a tumor suppressor by inhibiting cell proliferation and inducing apoptosis (393). However, as malignant cells acquire resistance to these growth-inhibitory effects, TGF-β transitions into a tumor-promoting factor, driving invasiveness, EMT, and metastatic dissemination (393). Within the TME, TGF-β1 secreted by TAMs induces EMT and enhances Liver-CSC traits (394). Furthermore, oncogenic cyclin D1 (CCND1) directly engages the SMAD machinery, forming a CCND1-SMAD2/3-SMAD4 complex that is essential for maintaining Liver-CSC stemness (395).

NOTCH signaling regulates Liver-CSC maintenance through juxtacrine communication, with pathway dysregulation promoting self-renewal, differentiation, tumorigenicity, angiogenesis, and migration (67, 396–398). The pathway is normally restrained by tumor suppressors including chromosome 8 open reading frame 4 (C8orf4)—which binds the NOTCH2 intracellular domain (N2ICD) to prevent nuclear translocation —and runt-related transcription factor 3 (RUNX3), which suppresses Jagged1-NOTCH signaling (398–400). Loss of these regulators unleashes NOTCH activity, enhancing Liver-CSC self-renewal, tumorigenicity, and metastatic potential. Furthermore, the inflammatory TME modulates NOTCH through inducible nitric oxide synthase (iNOS)-generated nitric oxide (NO), which enhances CD24^+^/CD133^+^ Liver-CSCs self-renewal via TNFα-converting enzyme (TACE/ADAM17)-dependent NOTCH1 activation (106, 401).

The Hh signaling pathway, a crucial developmental pathway, becomes pathologically activated in Liver-CSCs, driving their expansion (402). CD133^+^ Liver-CSCs demonstrate particularly upregulated Hh pathway activity (403). Mechanistically, the stress-inducible protein Sestrin3 normally constrains Hh signaling by binding to glioma-associated oncogene homolog 2 (GLI2) and inhibiting its nuclear translocation; Sestrin3 deficiency consequently promotes HCC pathogenesis through enhanced Hh signaling (404). This clinical significance is underscored by the correlation between reduced Sestrin3 expression and diminished patient survival, while Sestrin3 knockout mice develop increased tumor burden with elevated CD133/CD44 expression in diethylnitrosamine/choline-deficient high-fat diet (DEN/CD-HFD) models (404).

Hippo pathway dysregulation represents a fundamental mechanism in Liver-CSC maintenance. Pathway inactivation leads to YAP1/TAZ nuclear accumulation, where they interact with multiple oncogenic pathways (NOTCH, MAPK, Wnt) to drive stemness, EMT, drug resistance, and tumor progression (405–408). YAP1 upregulation in liver cancer cells promotes the CSC phenotype and correlates with elevated expression of stemness markers NANOG, OCT4, and CD133 (409).

In summary, diverse signaling pathways converge on a common function: to enforce the self-renewing, therapy-resistant state of Liver-CSCs. This network represents a critical lynchpin in HCC pathogenesis and a promising target for therapeutic intervention.

### 8.2 Epigenetic regulation

Epigenetic mechanisms—including DNA methylation, histone modifications, non-coding RNA regulation, and RNA methylation—play a pivotal role in maintaining the plasticity of Liver-CSCs. These reversible modifications provide a dynamic regulatory framework that allows Liver-CSCs to adapt rapidly to changing microenvironments and therapeutic pressures (61, 410, 411).

DNA methylation is a key epigenetic modification that regulates gene expression in Liver-CSCs. For example, in CD133- Huh7 cells, TGF-β1 signaling suppresses the DNA methyltransferases DNMT1 and DNMT3β. This inhibition leads to specific demethylation of the CD133 promoter-1, resulting in CD133 upregulation. Critically, this epigenetically reprogrammed CD133^+^ population demonstrates enhanced tumorigenicity, thereby reinforcing stem-like properties (412).

Histone modifications critically regulate Liver-CSC properties through a concerted program of transcriptional repression that maintains the stem cell state. Key demethylase and deacetylase enzymes work to silence differentiation genes and tumor suppressor pathways. Specifically, the histone deacetylase 3 (HDAC3) is selectively overexpressed in Liver-CSCs, where it compacts chromatin to repress differentiation genes while supporting the expression of core pluripotency factors like NANOG, OCT4, and SOX2 (413). In a parallel mechanism, the histone lysine demethylase LSD1 is upregulated in LGR5^+^ HCC cells and removes activating H3K4me1/2 marks from the promoters of key Wnt/β-catenin inhibitors, Prickle1 and APC. This epigenetic repression leads to β-catenin activation, thereby driving the self-renewal, tumorigenicity, and chemoresistance of LGR5^+^ Liver-CSCs (80).

Non-coding RNAs, including miRNAs, lncRNAs, and circRNAs, are central regulators of Liver-CSCs. Dysregulated miRNAs, such as miR-HCC2 (414), ​​miR-217​​ (415), miR-452 (416), miR-1246 (417), and miR-5188 (418), collectively enhance stem-like traits in HCC cells by modulating the Wnt/β-catenin signaling pathway. Likewise, circRNAs, particularly rtcisE2, are highly expressed in Liver-CSCs and also promote stemness through Wnt/β-catenin pathway activation (419). Furthermore, numerous lncRNAs—such as TCF7 (104), β-Catm (420), FZD6 (421), SNHG5 (422), NUTM2A-AS1 (423), DANCR (424), TIC1 (425), FOXD2-AS1 (426), LINC00662 (427), LINC00346 (428), DLGAP1-AS1 (429), SOX9-AS1 (430), OTUD6B-AS1 (431), and FEZF1-AS1 (432)—are overexpressed in Liver-CSCs and drive stemness, proliferation, invasion, EMT, and metastasis via Wnt pathway activation. Other lncRNAs, such as LncHDAC2 and circIPO11, fuel self-renewal and maintenance by activating Hh signaling (433, 434). In a separate mechanism, lncBRM recruits the brahma-related gene (BRM) to the switch/sucrose non-fermentable (SWI/SNF) chromatin remodeling complex, activating YAP1 signaling to maintain Liver-CSC stemness and tumor-initiating capacity (435), while lncRNA MALAT1 enhances Liver-CSC properties by sponging miR-375, which derepresses YAP1 expression (436).

RNA methylation, through modifications like N^6^-methyl-adenosine (m^6^A) and N¹-methyl-adenosine (m¹A), is a critical regulator of Liver-CSC maintenance and therapy resistance. For instance, the m^6^A writer methyltransferase-like 3 (METTL3) stabilizes key messenger RNAs (mRNAs) such as SOCS3 and LRP6, enhancing JAK2/STAT3 and Wnt/β-catenin signaling, respectively (437). Crucially, METTL3-mediated m^6^A modification upregulates FZD10 mRNA; in turn, FZD10 promotes liver CSC self-renewal, tumorigenicity, and lenvatinib resistance by co-activating the β-catenin and YAP1 signaling pathways (438). In a parallel mechanism, the tRNA methyltransferase 6/tRNA methyltransferase 61A (TRMT6/TRMT61A) complex catalyzes m¹A on tRNA-Arg-CCG, which boosts cholesterol synthesis to fuel Hh signaling (439).

In summary, the dynamic interplay of epigenetic mechanisms underpins the plasticity, adaptability, and resilience of Liver-CSCs. Consequently, targeting these epigenetic pathways presents a promising strategy for eradicating Liver-CSCs and improving HCC treatment outcomes.

### 8.3 Metabolic reprogramming

Metabolic reprogramming is a hallmark of Liver-CSCs, essential for fulfilling their heightened bioenergetic and biosynthetic demands, which underpin proliferation, survival, therapy resistance, and immune evasion (440). This reprogramming involves a shift in energy pathways. While non-CSCs are typically glycolytic, Liver-CSCs rely more heavily on mitochondrial respiration to maintain stemness, even as retrodifferentiation of HCC cells into this state is accompanied by signs of compromised mitochondrial function, such as diminished membrane potential, reduced ATP synthesis, and increased lactate production (441). The SIRT1/mitochondrial ribosomal protein S5 (MRPS5) axis is central to this metabolic network; MRPS5 generates nicotinamide adenine dinucleotide (NAD^+^), and SIRT1, enriched in Liver-CSCs, post-translationally regulates MRPS5 via deacetylation (442). Elevated SIRT1 and cytoplasmic MRPS5 correlate with poor HCC prognosis, positioning this axis as a therapeutic target (442). Paradoxically, enhanced glycolysis and MYC upregulation also drive Liver-CSC proliferation in certain contexts, revealing a complex and adaptable metabolic landscape (443).

Lipid metabolism is profoundly augmented in Liver-CSCs. They demonstrate enhanced fatty acid metabolism, vital for self-renewal, tumorigenicity, and therapeutic persistence (443, 444). This process involves *de novo* lipogenesis, promoting lipid accumulation that serves as both energy reservoirs and structural components for membranes, thereby reinforcing stemness and apoptotic resistance (443, 445, 446). Key orchestrating enzymes include fatty acid synthase (FASN) and stearoyl-CoA desaturase 1 (SCD1) (443, 445, 446). Furthermore, Liver-CSCs preferentially utilize fatty acid oxidation (FAO) over oxidative phosphorylation, a metabolic switch indispensable for sustaining stemness and therapy resistance (447, 448). This shift holds particular relevance in NANOG-mediated stemness and MASLD-related HCC etiology (306, 307). Lipid droplets function as auxiliary energy sources during glycolytic inhibition, exemplifying metabolic flexibility (445, 449). This lipid metabolism is regulated by Wnt/β-catenin signaling and peroxisome proliferator-activated receptors (PPARs), which promote Liver-CSC traits (444, 445). Therapeutically, FASN inhibitors (e.g., cerulenin) attenuate CSC properties and synergize with sorafenib (449, 450).

Beyond fatty acid metabolism, Liver-CSCs display hyperactive cholesterol biosynthesis. Here, caspase-3-mediated activation of sterol regulatory element-binding protein 2 (SREBP2) accelerates cholesterol production, promoting proliferation, conferring sorafenib/lenvatinib resistance, and activating Hh signaling to cause therapeutic refractoriness (439). This oncogenic pathway is reinforced by the upregulation of 3-hydroxy-3-methylglutaryl-coenzyme A reductase (HMGCR), a key cholesterol synthesis enzyme that also promotes Liver-CSC stemness and metastasis via Hh signaling (451).

The pentose phosphate pathway (PPP) is indispensable for generating nicotinamide adenine dinucleotide phosphate (NADPH) to maintain redox balance and ribose-5-phosphate for nucleotide synthesis, supporting the high replication rate of rapidly dividing Liver-CSCs and their survival in a challenging TME (452). Clinically, aberrant expression of PPP enzymes—notably glucose-6-phosphate dehydrogenase (G6PD)—represents an emerging biomarker for HCC diagnosis and prognosis (453–455).

Reprogramming of amino acid metabolism is equally critical. Liver-CSCs show significant upregulation of tyrosine, alanine, and glutamine pathways (456, 457). Chemoresistant Liver-CSCs exhibit greater dependence on glutamine than glucose, a vulnerability mediated by glutaminase 1 (GLS1)—which catalyzes the conversion of glutamine to glutamate—positioning GLS1 inhibition as a promising therapeutic strategy (313, 314). Notably, these cells maintain stemness even under glutamine deprivation, revealing profound metabolic plasticity (458). CD13^+^ Liver-CSCs exploit tyrosine metabolism to generate nuclear acetyl-CoA, acetylating and stabilizing FOXD3 to enforce quiescence and chemoresistance (459). Parallelly, alanine-glyoxylate aminotransferase (AGXT) sustains a serine-glycine-alanine metabolic axis that directly upregulates core pluripotency factors SOX2 and OCT4 (460).

Integral to these networks, ROS homeostasis is a fundamental component of Liver-CSC metabolism, functioning as both metabolic byproducts and dynamic regulators of stemness. While low ROS facilitate proliferative signaling, pathological accumulation induces oxidative damage (461–464). Liver-CSCs consistently exhibit enhanced antioxidant capacity and reduced ROS levels, potentially linked to NRF2 activation, which preserves self-renewal and therapy resistance (465). NRF2 activation enhances HIF-1α expression, driving metabolic reprogramming that preserves Liver-CSC stemness (466, 467). Crucially, ROS balance fundamentally determines Liver-CSC stemness. Increased GLS1 activity reduces intracellular ROS, which amplifies β-catenin-mediated transcription of Wnt target genes, upregulating essential stemness markers (CD44, SOX2, OCT4) (78, 456).

To summarize, these metabolic adaptations—reveal druggable vulnerabilities in Liver-CSC metabolism, offering strategic targets to impede HCC progression.

## 9 Role of Liver-CSCs in current therapy resistance and rationale for their therapeutic targeting in HCC

**Figure 4:**
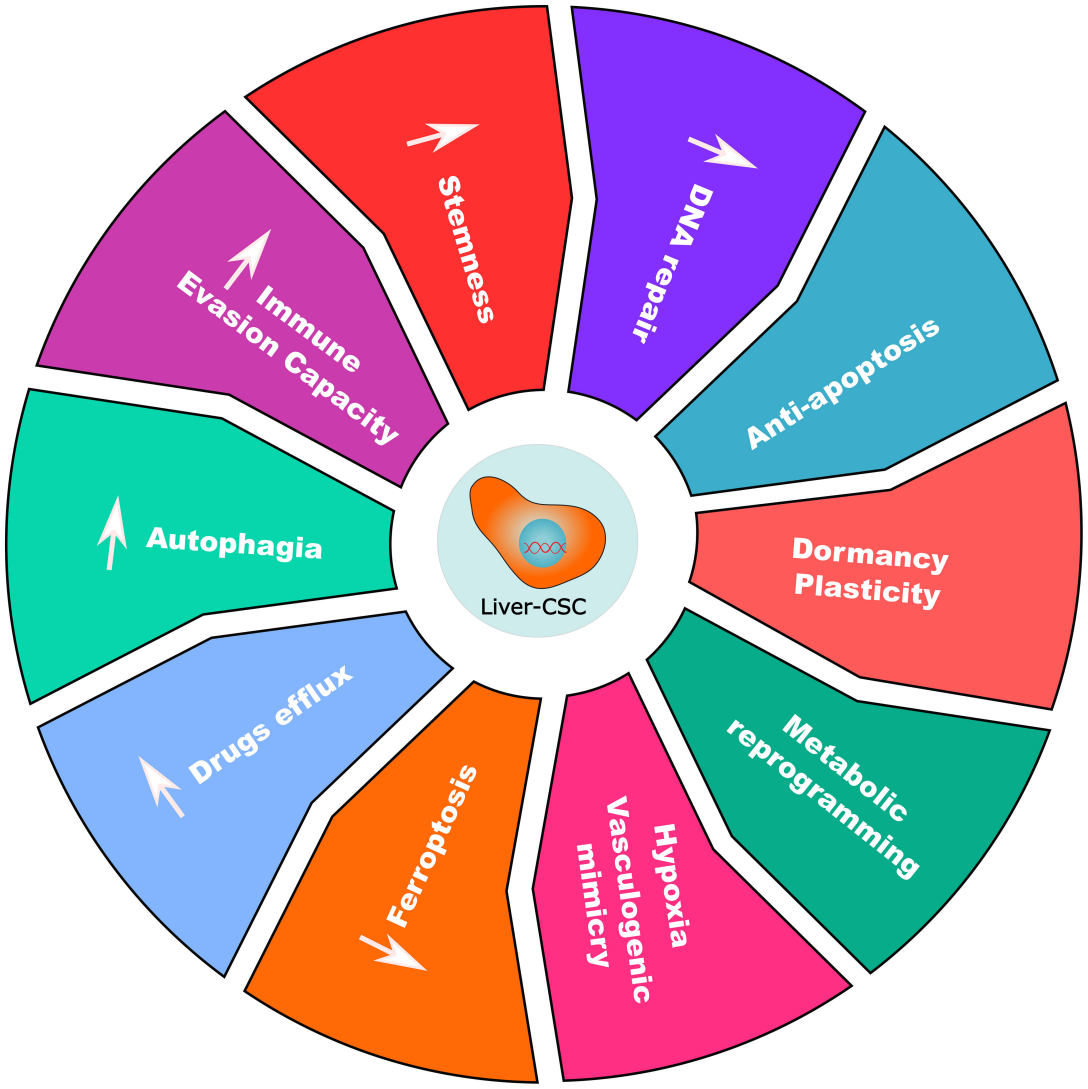
Liver-CSC resistance mechanisms. Liver-CSCs evade treatment through: dormancy and plasticity; hypoxia and vasculogenic mimicry; enhanced stemness; autophagy; anti-apoptosis; enhanced drug efflux; enhanced DNA repair capacity; metabolic reprogramming; enhanced immune evasion capacity; and reduced ferroptosis. Following survival, these cells drive tumor repopulation, progression, recurrence, and metastasis.

Liver-CSCs drive therapy resistance in HCC through diverse mechanisms (**Figure 4**). This section delineates their pathophysiological role and synthesizes the rationale for molecularly targeted interventions against these resilient cells.

### 9.1 Residual Liver-CSCs and dormancy

While liver resection and transplantation effectively remove macroscopic tumor masses, they cannot eliminate microscopic residual disease harboring Liver-CSCs. These cells may persist as minimal residual disease (MRD) in the liver remnant or circulate as CTCs (19, 210, 468), acting as reservoirs for recurrence. Similarly, radiofrequency ablation (RFA) aims to eradicate cancer cells through high-temperature-induced cellular injury (469). However, the “heat-sink” effect from adjacent vasculature often reduces RFA efficacy, leading to incomplete ablation of the tumor cells and leaving behind residual Liver-CSCs that drive tumor recurrence (470, 471).

Under genotoxic stress from therapies that target rapidly dividing cells, such as chemotherapy and radiotherapy, Liver-CSCs enter a dormant state. This allows them to evade therapy by maintaining genomic stability and metabolic resilience. These quiescent cells act as biological “seeds,” regenerating tumors upon reactivation (472–474). CD13 marks quiescent Liver-CSCs, countering genotoxic stress by scavenging ROS and mitigating DNA damage (49). CD13 further promotes HCC progression and sorafenib resistance by stabilizing p65 via HDAC5 interaction, hyperactivating NF-κB signaling (475).

### 9.2 Hypoxia

Building upon the foundational role of hypoxia within the Liver-CSC niche in driving aggressive Liver-CSC phenotypes (344, 355), emerging evidence underscores that conventional anticancer therapies paradoxically amplify intratumoral hypoxia, thereby fortifying the resistant Liver-CSC populations they intend to eliminate.

This therapy-induced hypoxia arises through multiple iatrogenic mechanisms. In the surgical context, post-resection hypoxia can result from vascular occlusion (e.g., Pringle’s maneuver), cold ischemia during transplantation, or compromised perfusion in the regenerating liver remnant (476–478). Similarly, non-surgical interventions directly create hypoxic conditions that favor Liver-CSC enrichment. TACE acts by design, inducing ischemic necrosis through the blockade of tumor-feeding vessels (479, 480). Ablation therapies generate zones of intense local hypoxia surrounding the areas of thermal tissue destruction (481, 482). Furthermore, both chemotherapy and radiotherapy compromise the tumor stroma and microvasculature, causing hypoxia that actively drives the evolution of more aggressive, therapy-resistant Liver-CSCs (483, 484).

Consequently, therapy-induced hypoxia may represent a pivotal, self-reinforcing mechanism of Liver-CSC-driven resistance. By reshaping the TME, common anticancer therapies may inadvertently expand the resistant Liver-CSC pool. This hypoxia-driven resistance likely synergizes with dormancy programs and stemness pathways, creating a multifaceted defense network in HCC. This cycle underscores the critical need for therapeutic strategies that co-target the hypoxic Liver-CSC niche to overcome treatment resistance.

### 9.3 Enhanced stemness

Enhanced stemness critically underpins Liver-CSC-mediated therapy evasion and recurrence in HCC. Following sub-lethal RFA, Liver-CSCs significantly upregulate heat shock proteins (HSPs), which function as molecular chaperones to mitigate heat-induced proteotoxicity and cellular damage, thereby enhancing survival and therapeutic resistance (357, 485, 486),. Wan et al. demonstrated that incomplete RFA promotes malignant progression via FOXP4-mediated induction of N-deacetylase and N-sulfotransferase 2 (NDST2), where FOXP4 knockdown suppresses tumor progression and NDST2 expression in residual HCC (487). Strikingly, elevated FOXP4 expression correlates with enhanced CSC stemness and metastasis in gastric cancer (488), supporting its conserved role in Liver-CSC-driven recurrence. Similarly, RFA accelerates recurrence through VEGF release, activating VEGFR2 on CD133^+^ Liver-CSCs to trigger NANOG-mediated self-renewal programs and amplify stem-like properties (69).

Concurrently, chronic HBV infection—a major HCC inducer—impairs sorafenib response (489, 490), with clinical trials confirming poor outcomes in HBV-positive patients (491). The hepatitis B virus X protein (HBx) and surface antigen (HBsAg) enhance Liver-CSC stemness and tumorigenicity by upregulating core pluripotency factors (c-MYC, KLF4, NANOG, OCT4, SOX2), surface markers (CD90, CD117, CD133), and dysregulating oncogenic miRNAs through upregulation of miR-181 and downregulation of tumor-suppressive miR-203a (492, 493). Critically, HBx activates the MDM2/CXCL12/CXCR4/β-catenin signaling axis in OV6^+^ Liver-CSCs, driving stemness amplification and intrinsic sorafenib resistance (494). HCV infection similarly drives stemness-mediated therapy evasion. It induces core stemness regulators (DCAMKL-1, LGR5, CD133, AFP, CK19, Lin28, c-MYC), establishing chemoresistant tumor-initiating phenotypes (495).

Emerging research identifies FZD10 and phosphorylated non-muscle myosin heavy chain 9 (pMYH9) overexpression in Liver-CSCs as novel mediators of lenvatinib resistance, where these proteins enhance stemness while activating Wnt/β-catenin, Hippo, and HIF-1α pathways (496, 497). Similarly, serine protease inhibitor kazal type 1 (SPINK1) has been implicated in chemoresistance in HCC (498, 499). SPINK1 is enriched in CD133^+^ HCC cells and promotes stemness, dedifferentiation, and chemoresistance to sorafenib, oxaliplatin, 5-fluorouracil (5-FU), and cisplatin by activating the ERK-CDK4/6-E2F2 signaling cascade (499). Other proteins, including minichromosome maintenance complex component 2 (MCM2) through the Hippo pathway (500), receptor for activated C kinase 1 (RACK1) through NOTCH signaling (501), transcription factor CP2-like 1 (TFCP2L1) through the NANOG/STAT3 axis (502), and abelson interactor 2 (ABI2) through the MEOX2/KLF4-NANOG pathway (503), have also been linked to Liver-CSC stemness and sorafenib resistance.

### 9.4 Enhanced autophagy

Autophagy serves as a critical survival mechanism enabling Liver-CSCs to evade therapy and drive HCC progression. It directly mediates tyrosine kinase inhibitor (TKI) resistance through CD24 upregulation, which induces protein phosphatase 2A (PP2A)-mediated downregulation of mTOR/AKT signaling and promotes pro-survival autophagy (504). Concurrently, annexin A3 (ANXA3) overexpression induces autophagy in CD133^+^ Liver-CSCs, enhancing cellular survival and conferring resistance to sorafenib and regorafenib (505–507).

Critically, autophagy pathways are also activated under therapeutic stress from ablation procedures. Incomplete RFA triggers activation of autophagy (357, 481), initiated via HIF-1α/BCL2 interacting protein 3 (BNIP3) and HGF/c-Met pathways—promotes residual cancer cell survival, accelerating post-treatment recurrence, proliferation, and invasiveness (508, 509). Recurrent HCC following RFA demonstrates upregulated Liver-CSC markers (CD133, EpCAM) (509), with Wang et al. confirming autophagy increases HCC cell viability and invasiveness post-RFA, where CD133 facilitates autophagosome formation (510). Clinically, HCC patients undergoing thermal ablation show incomplete ablation correlates with elevated tumor progression risk and significantly reduced progression-free survival (PFS) compared to complete responders (357, 511).

Taken together, these results demonstrate that autophagy is among the key mechanisms enabling Liver-CSCs in HCC to resist therapy, thereby supporting their survival and contributing to tumor progression.

### 9.5 Anti-apoptosis

Liver-CSCs evade therapeutic elimination through robust anti-apoptotic mechanisms. These cells overexpress apoptotic inhibitors—including PI3K, Cdc-42, caspase 3, NF-κB, Bcl-2, Bad-p, Mcl-1, and Bax—which collectively prevent mitochondrial cytochrome c release and apoptosis initiation (512–514). Resistance to sorafenib is further driven by upregulation of anti-apoptotic genes inhibitor of apoptosis protein 2 (cIAP2), survivin, and X-linked inhibitor of apoptosis protein (XIAP) (489, 515–517). Paradoxically, chemotherapeutic agents like sorafenib can activate the anti-apoptotic transcription factor NF-κB (518), with evidence confirming sorafenib-induced NF-κB activation enhances resistance specifically in CD133^+^ Liver-CSCs (519).

CD133^+^ Liver-CSCs exhibit pronounced anti-apoptotic and radioresistant properties. Following radiation, these cells display enhanced MAPK/PI3K pathway activation and significantly reduced ROS levels compared to CD133^-^ cells (131). *In vivo* validation in xenograft models demonstrated that irradiated CD133^+^ cells generated significantly more tumors in nude mice than irradiated CD133^-^ cells, establishing their critical role in HCC radioresistance (131). Additionally, high expression of the scaffolding protein 14-3-3ζ in Liver-CSCs regulates apoptosis, differentiation, and cell cycle progression. Depletion of 14-3-3ζ increases radiosensitivity, likely by impairing apoptosis suppression (520).

### 9.6 Enhanced drug efflux

Elevated expression of ABC transporters—including multidrug resistance protein 1 (MDR1/ABCB1), multidrug resistance-associated protein 1 (MRP1/ABCC1), and breast cancer resistance protein (BCRP/ABCG2)—in Liver-CSCs actively effluxes cytotoxic agents. This reduces intracellular drug accumulation, conferring broad resistance to chemotherapeutics and targeted therapies (86). Notably, inhibition of ABCB1/MDR1 and ABCG2/BCRP in EpCAM^+^–CD133^+^ Huh7 and PLC/PRF/5 cells using pharmacological inhibitors or RNA interference (RNAi) has been shown to increase intracellular doxorubicin concentration, suggesting a promising strategy to enhance chemotherapy efficacy, particularly in patients undergoing TACE (521). Li et al. demonstrated that that insulin-like growth factor II mRNA-binding protein 3 (IGF2BP3) overexpression in Liver-CSCs upregulates CD133 and ABC transporters, enhancing tumor sphere formation and conferring resistance to sorafenib and doxorubicin (522). Critically, high IGF2BP3 expression independently predicts poor survival in HCC patients, underscoring its clinical relevance (522).

### 9.7 Enhanced DNA repair capacity

Liver-CSCs demonstrate heightened resistance to chemotherapy and radiotherapy due to superior DNA repair capabilities compared to non-CSCs (131, 523, 524). This resilience is driven by overexpression of key DNA repair proteins: chromodomain helicase DNA binding protein 4 (CHD4), ataxia telangiectasia mutated (ATM), ataxia telangiectasia and rad3-related (ATR), checkpoint kinases 1 and 2 (Chk1/Chk2), poly(ADP-ribose) polymerase-1 (PARP-1), DNA polymerase beta (POLβ), the XRCC1-DNA ligase IIIa complex, MRE11-RAD50-NBS1 (MRN) complex, and breast cancer 1 (BRCA1) (525–528). These proteins collectively detect and repair DNA damage while accelerating restoration of genomic integrity, effectively neutralizing cytotoxic therapies. Crucially, their overexpression simultaneously reinforces CSC properties. The intricate coordination within this repair network highlights both its biological complexity and therapeutic vulnerability (497–500).

### 9.8 Metabolic adaptations

As discussed earlier metabolic reprogramming constitutes a core adaptive strategy in CSCs (440), with ALDH enzymes serving as pivotal drivers of chemoresistance (83, 529). These enzymes protect CSCs through detoxification mechanisms: converting cytotoxic aldehydes (e.g., malondialdehyde, hydroxy-hexenal, and 4-hydroxy-2-nonenal (4-HNE) generated by chemo/radiotherapy) into inert carboxylic acids while simultaneously reducing oxidative stress (529, 530). This aldehyde clearance shields against DNA damage and suppresses immunogenic cell death, potentially dampening antitumor immunity through effective clearance of ROS (529–532). Beyond detoxification, specific ALDH isoforms (ALDH1A1/ALDH3A1) actively metabolize chemotherapeutic agents—including cyclophosphamide analogs like ifosfamide and etoposide—by inactivating intermediates such as aldophosphamide into carboxyphosphoramide (533, 534). ALDH further maintains CSC homeostasis by synthesizing retinoic acid and γ-aminobutyric acid (GABA), thereby regulating differentiation through NOTCH, mTOR, and PI3K/AKT pathways (535–537). Clinically, ALDH inhibition (e.g., via all-trans retinoic acid) reverses therapy resistance across diverse malignancies; however, its specific role in Liver-CSC drug evasion remains an active research frontier, with targeting strategies holding promise for sensitizing refractory tumors to conventional therapies (84, 538).

### 9.9 Enhanced immune evasion capacity

Liver-CSCs frequently overexpress PD-L1 compared to non-CSCs within tumors, contributing to their immune evasion (539). This overexpression is regulated by lysosome-associated protein transmembrane 4 beta (LAPTM4B), controlled transcriptionally by ETV1 through the Wnt1/c-MYC/β-catenin signaling pathway (540). By engaging PD-1 receptor on CD8^+^T cells, Liver-CSC-derived PD-L1 induces T cell exhaustion and suppresses antitumor activity, manifesting as diminished T-cell proliferation and IFN-γ production (541–543). Mechanistically, Kong et al. demonstrated that PD-L1 overexpression in CD133^+^ Liver-CSCs enhances stem-like properties, tumor sphere formation, EMT, and invasiveness through the serum- and glucocorticoid-inducible kinase 2 (SGK2)/β-catenin pathway (544). Clinically, PD-L1 overexpression in post-resection HCC specimens from sorafenib-treated patients independently predicts recurrence and correlates with significantly worse RFS (545). Paradoxically, although PD-L1^high^ Liver-CSCs should exhibit susceptibility to PD-L1-targeting immune checkpoint inhibitors, some patients with PD-L1^high^ tumors fail to respond (539, 546), highlighting a key knowledge gap in immunotherapy response dynamics.

Beyond PD-L1 dysregulation, Liver-CSCs drive HCC immune evasion by producing immunosuppressive cytokines (TGF-β, IL-10) that directly suppress T cells, thereby evading immune-mediated destruction (547, 548). Additionally, Liver-CSCs actively recruit immunosuppressive cells (MDSCs, Tregs, TAMs, and TANs) through signaling mechanisms and cytokine release, fostering an immunosuppressive TME. This skews immune responses toward immunosuppression, potentiating Liver-CSCs-mediated immune evasion and immunotherapy resistance (549–551).

Furthermore, Liver-CSCs downregulate major histocompatibility complex class I (MHC-1) expression (549), impairing antigen presentation and evading immune surveillance (552, 553). This evasion subverts T cell-mediated cytotoxicity, thereby underpinning immunotherapy resistance. Recent research reveals that phosphatase and tensin homolog deleted on chromosome 10 (PTEN) deficiency in CSCs, including Liver-CSCs, reduces neoantigen expression and contributes to immunotherapy resistance (554, 555). Similarly, Galarreta et al. demonstrated that β-catenin activation in HCC promotes resistance to anti-PD-1 therapy by enabling immune escape (556). This aligns with earlier evidence that β-catenin activation activates Liver-CSCs (557), suggesting β-catenin may contribute to PD-L1 inhibitor resistance via Liver-CSCs.

### 9.10 Dynamic plasticity of Liver-CSCs

The plasticity of Liver-CSCs fundamentally challenges the traditional static hierarchical view of tumor cell populations. his model posits that tumor cells can reversibly switch between non-CSC and Liver-CSC states—a dynamic process driven by environmental and therapeutic pressures. This phenotypic plasticity is a central mechanism underlying intratumoral heterogeneity and therapeutic failure in HCC (558–560).

The reversible processes of EMT and MET are key facilitators of this plasticity, allowing cells to navigate a spectrum of states. Within this spectrum, a “stemness window” emerges—a transient, hybrid epithelial/mesenchymal (E/M) state that confers maximal tumor-initiating potential. This state is not a permanent cell fate but a temporary, high-plasticity condition modulated by molecular and microenvironmental signals (561–563).

The molecular architecture of this plasticity involves sophisticated regulatory networks. Core pluripotency factors (OCT4, SOX2, NANOG) coordinate with EMT regulators (ZEB1, SNAIL) through integrated signaling pathways including TGF-β, Wnt/beta-catenin, Hh, and NOTCH (558, 564). The TME actively maintains this plastic state through stromal interactions, particularly CAFs operating via the c-Met/FRA1/HEY1 axis (224). The link between this molecular machinery and the stem-like phenotype is well-established. Research indicates that cell surface vimentin (csVim) serves as a functional marker to identify and isolate cells with Liver-CSC properties, where csVim^+^CD133^-^ populations display stemness characteristics comparable to traditional CD133^+^ Liver-CSCs (565). Furthermore, defined Liver-CSC subsets, such as CD90^+^ and CD44^+^ cells, consistently exhibit a molecular profile indicative of EMT—characterized by elevated levels of mesenchymal markers like N-cadherin and vimentin, coupled with a reduction in the epithelial marker E-cadherin (566, 567).

Clinical evidence demonstrates that therapeutic interventions paradoxically enhance this plasticity. Sorafenib induces epigenetic reprogramming through capicua inactivation and ERK-mediated expansion of EpCAM^+^ Liver-CSCs (560). Similarly, genotoxic stress from chemotherapy triggers *de novo* generation of CD90^+^ and CD105^+^ mesenchymal subsets (568), confirming that treatments does not merely select for pre-existing resistant clones but actively creates them, fundamentally reshaping tumor heterogeneity.

This paradigm dictates that targeting a static Liver-CSC population is insufficient. Durable responses require strategies that lock cells in a non-tumorigenic state by targeting the plasticity process itself. This includes disrupting the drivers of plasticity, inducing MET to abolish stemness, or forcing terminal differentiation of Liver-CSCs.

## 10 Therapeutic strategies and targets for Liver-CSCs

Current drug development targets Liver-CSCs through three primary approaches: 1) surface markers, 2) core signaling pathways, and 3) niche disruption, with emerging strategies expanding this arsenal. The following sections will explore these major strategies, along with others, in depth.

### 10.1 Targeting Liver-CSC markers

**Figure 5:**
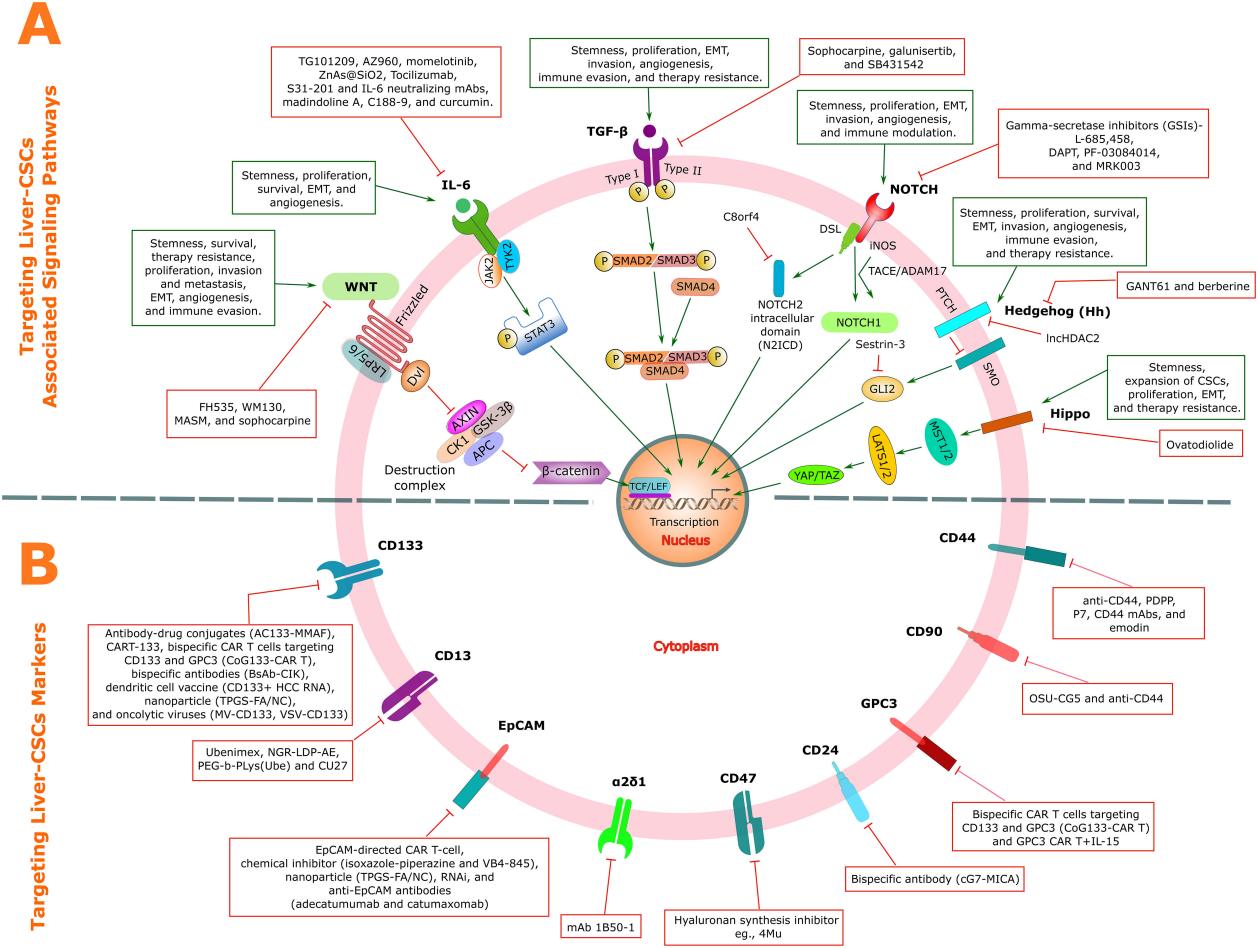
Liver-CSC therapeutic strategies. **(A)** Targeting signaling pathways: WNT pathway, IL-6/JAK/STAT signaling pathway, TGF-β/SMAD signaling pathway, NOTCH signaling pathway, Hedgehog signaling pathway, Hippo signaling pathway. **(B)** Targeting markers: CD133, CD13, EpCAM, α2δ1, CD47, CD24, GPC3, CD90, CD44. *Red box: Therapies targeting pathways/markers. Green box: Pathway-mediated enhancement of Liver-CSC properties.*.

Therapeutic strategies targeting Liver-CSC-specific markers (e.g., CD133, EpCAM, CD90) represents a strategic approach against HCC, utilizing diverse modalities including monoclonal antibodies (mAbs), chimeric antigen receptor T (CAR-T) cells, bispecific antibodies (BsAbs), dendritic cell vaccines, peptides, and oncolytic viruses (**Figure 5**). Corresponding clinical trials are detailed in **Table 2**.

**Table 2:** Clinical trials targeting liver-CSCs markers.

Marker	NCT number	Conditions	Interventions	Enrollment	Locations	Phases	Study status	Study results
Epithelial cell adhesion molecule (EpCAM)								
	NCT03013712	Solid malignancies (including hepatocellular carcinoma)	CAR-T cell immunotherapy	60	China	Phase 1/2	Unknown	No
	NCT05028933	Advanced digestive system malignancies (including hepatocellular carcinoma)	EPCAM CAR-T	48	China	Phase 1	Recruiting	No
	NCT02729493	Liver neoplasms	EPCAM-targeted CAR-T cells	25	China	NA	Unknown	No
CD133								
	NCT02541370	Various cancers (including hepatocellular carcinoma)	Anti-CD133-CAR vector-transduced T cells	20	China	Phase 1/2	Completed	No
Intercellular adhesion molecule-1 (ICAM-1, CD54)								
	NCT00028496	Solid malignancies (including hepatocellular carcinoma)	Recombinant fowlpox-CEA(6D)/TRICOM vaccine, sargramostim, recombinant fowlpox GM-CSF vaccine adjuvant	48	United States	Phase 1	Completed	No
C-kit (CD117)								
	NCT04476329	Hepatocellular carcinoma	Regorafenib 40 MG	7	United States	Phase 2	Terminated	No
	NCT00427973	Hepatocellular carcinoma	Cediranib maleate (AZD2171)	17	United States	Phase 2	Terminated	Yes
	NCT04479527	Hepatocellular carcinoma	Camrelizumab	34	China	Phase 2	Unknown	No
Glypican-3 (GPC3)								
	NCT03130712	Hepatocellular carcinoma	GPC3-CART cells	10	China	Phase 1/2	Unknown	No
	NCT02715362	Hepatocellular carcinoma	TAI-GPC3-CART cells	30	China	Phase 1/2	Unknown	No
	NCT05047510	Hepatocellular carcinoma	Anti-GPC3-IRDye800CW	60	China	NA	Recruiting	No
	NCT03084380	Hepatocellular carcinoma	Retroviral vector-transduced autologous T cells to express anti-GPC3 CARs, Fludarabine, Cyclophosphamide	20	China	Phase 1/2	Unknown	No
	NCT02723942	Hepatocellular carcinoma	CAR-T cell immunotherapy	0	China	Phase 1/2	Withdrawn	No
	NCT06383520	Malignant liver neoplasms	68Ga-aGPC3-scFv/Fab	100	China	Early Phase 1	Recruiting	No
	NCT03198546	Hepatocellular carcinoma and lung cancer	GPC3 and/or TGFβ targeting CAR-T cells	30	China	Phase 1	Recruiting	No
	NCT05003895	Hepatocellular carcinoma	Cyclophosphamide, CAR-T cell, Fludarabine	38	United States	Phase 1	Recruiting	No
	NCT04121273	Hepatocellular carcinoma	CAR-T cell immunotherapy	20	China	Phase1	Unknown	No
	NCT06641453	Hepatocellular carcinoma	GPC3-CART cells, Fludarabine Phosphate, Cyclophosphamide	30	China	Phase 1/2	Not yet recruiting	No
	NCT02395250	Hepatocellular carcinoma	GPC3 CAR T	13	China	Phase 1	Completed	No
	NCT04506983	Hepatocellular carcinoma	GPC3-CAR-T cells	12	China	Phase 1	Suspended	No
	NCT05352542	Hepatocellular carcinoma	GPC3 Targeting CART Cells	10	China	Phase 1	Terminated	No
	NCT05620706	Hepatocellular carcinoma	GPC3 CAR-T cells	20	China	NA	Recruiting	No
	NCT06590246	Hepatocellular carcinoma	Armored and GPC3-targeted autologous CAR T-cell	121	China	Phase 1/2	Not yet recruiting	No
	NCT03146234	Hepatocellular carcinoma	CAR-GPC3 T cells	7	China	NA	Completed	No
	NCT03980288	Hepatocellular carcinoma	CAR-GPC3 T Cells	6	China	Phase 1	Completed	No
	NCT05344664	Not specified	GPC3-CAR-T cells	12	China	Phase 1	Not yet recruiting	No
	NCT04951141	Hepatocellular carcinoma	anti-GPC3 CAR-T cells	10	China	Early Phase 1	Unknown	No
	NCT03884751	Hepatocellular carcinoma	CAR-GPC3 T Cells	9	China	Phase 1	Completed	No
	NCT06560827	Hepatocellular carcinoma	CT011 CAR-GPC3 T Cells Injection	30	China	Phase 1	Recruiting	No
	NCT06198296	Various cancers (including hepatocellular carcinoma)	21.15.GPC3-CAR T cells	21	United States	Phase 1	Not yet recruiting	No
	NCT03175705	Hepatocellular carcinoma	HCC antigens-specific CD8+ T lymphocytes, IL-2, Tegafur	18	China	Phase 1	Unknown	No
	NCT06652243	Hepatocellular carcinoma	SN301A	12	China	Early Phase 1	Recruiting	No
	NCT05103631	Solid tumors (including hepatocellular carcinoma)	CATCH T cells	27	United States	Phase 1	Recruiting	No
	NCT05926726	Hepatocellular carcinoma	CAR-GPC3 T cells	12	China	NA	Recruiting	No
	NCT02905188	Hepatocellular carcinoma	GLYCAR T cells, Cytoxan, Fludarabine	9	United States	Phase 1	Completed	No
	NCT06144385	Hepatocellular carcinoma	CAR-GPC3 T cells	20	China	Phase 1	Recruiting	No
	NCT05652920	Hepatocellular carcinoma	Ori-C101	105	China	Phase 1/2	Recruiting	No
	NCT04093648	Hepatocellular carcinoma	TEGAR T cells, Cytoxan, Fludarabine	0	United States	Phase 1	Withdrawn	No
	NCT06478693	Hepatocellular carcinoma	MT-303	48	Australia	Phase 1	Recruiting	No
	NCT06461624	Hepatocellular carcinoma	anti-GPC3 CAR-T	15	China	Phase 1	Recruiting	No
	NCT06687941	Solid tumors (including hepatocellular carcinoma)	AST-201	70	NA	Phase 1	Not yet recruiting	No
	NCT05155189	Hepatocellular carcinoma	C-CAR031, Lenvatinib, PD-1(L1) monoclonal antibody	44	China	Phase 1	Recruiting	No
	NCT06088459	Hepatocellular carcinoma	NWRD06	9	China	Phase 1	Recruiting	No
	NCT06084884	Hepatocellular carcinoma	AZD5851	94	United States	Phase 1/2	Recruiting	No
	NCT05783570	Hepatocellular carcinoma	EU307 CAR-T Cell	12	Korea	Phase 1	Recruiting	No
	NCT05070156	Hepatocellular carcinoma	B010-A	3	China	Early Phase 1	Active not recruiting	No
	NCT04756648	Hepatocellular carcinoma	CT0180 Cells	21	China	Phase 1	Recruiting	No
	NCT06427941	Solid tumors (including hepatocellular carcinoma)	BGB-B2033, Tislelizumab	140	Multi-country	Phase 1	Recruiting	No
	NCT04973098	Hepatocellular carcinoma	CT0181 Cells	13	China	Phase 1	Unknown	No
	NCT05059821	Hepatocellular carcinoma	Peptide cancer vaccine	10	Egypt	Phase 1	Unknown	No
	NCT06715839	Solid tumors (including hepatocellular carcinoma)	[68Ga]Ga-DOTA-H2D3, [18F]F-RESCA-RB14, [68Ga]Ga-NOTA-T4, [18F]F-RESCA-T4, [68Ga]Ga-NOTA-G5, [18F]F-RESCA-G5, [68Ga]Ga-NOTA-WWH347, [18F]F-RESCA-WWH347, [68Ga]Ga-NOTA-RND20, [18F]F-RESCA-RND20	400	China	NA	Recruiting	No
	NCT02959151	Solid tumors (including hepatocellular carcinoma)	CAR-T cell	20	China	Phase 1/2	Unknown	No
Delta-Like 1 Homolog (DLK1)								
	NCT06636435	Solid tumors (including hepatocellular carcinoma)	CBA-1205	50	Japan	Phase 1	Recruiting	No

CD133-directed strategies show significant promise. Smith et al. developed an antibody-drug conjugate (ADC) using the anti-CD133 mAb AC133 conjugated to the cytotoxic drug monomethyl auristatin F (MMAF). This AC133-MMAF ADC demonstrated potent anti-tumor responses and delayed tumor growth in Hep3B xenograft models (569). Clinically, a Phase II trial of CD133-directed CAR-T cells (CART-133) showed promising antitumor activity and a manageable safety profile in advanced HCC patients, with a median OS of 12 months (570). Further innovation includes CD133-specific CAR-T cells engineered with the sleeping beauty (SB) system to produce PD-1-scFv, which exhibited significant antitumor activity *in vitro* and *in vivo* (571).

Notably, combinatorial targeting amplifies effects. Wang et al. demonstrated that bispecific CAR T cells (CoG133-CAR) targeting CD133 and GPC3, delivered via minicircle DNA vectors, eradicated tumors and extended survival in mouse models without off-target toxicity (572). Separately, a Phase I trial of GPC3 CAR-T cells incorporating IL-15 showed a 33% objective response rate and 66% stable disease rate in solid tumors, including HCC, with significantly enhanced T-cell expansion (573). Beyond CAR-T, BsAbs offer alternative precision. BsAbs such as anti-CD3/anti-CD133 constructs binding to cytokine-induced killer cells (CIK) effectively target and kill CD133^high^ Liver-CSCs (574). Similarly, BsAb cG7-MICA simultaneously activates NK cells via MHC class I-related chain A (MICA)/NK cell receptor group 2 member D (NKG2D) engagement and targets CD24^+^ Liver-CSCs, enhancing immune surveillance (575). Dendritic cell vaccines and oncolytic viruses provide complementary approaches. CD133 RNA-loaded vaccines stimulate potent CD8^+^ T cell responses against CD133^+^ Liver-CSCs (576). Likewise, oncolytic viruses like measles virus (MV-CD133) and vesicular stomatitis virus (VSV-CD133) selectively eliminate CD133^+^ Liver-CSCs, with VSV-CD133 demonstrating superior oncolytic activity and intratumoral spread (>10^2^-fold larger infected area than MV-CD133), crucial for targeting sparse CD133^+^ populations (577).

EpCAM is another high-priority target, with several clinical trials (e.g., NCT03013712, NCT05028933) evaluating EpCAM-directed CAR-T therapies. Chemical inhibitors (e.g., isoxazole-piperazine analogues) (578) and nitidine chloride nanoparticles (TPGS-FA/NC) (579) reduce CD133^+^EpCAM^+^ cells and pluripotency markers. Vaccination with CD44/EpCAM peptide-primed dendritic cells enhances anti-tumor immunity and induces apoptosis (580). The inhibitor VB4–845 targeting EpCAM^+^ Liver-CSCs shows anti-tumor cytotoxicity, especially combined with 5-FU (581). RNAi targeting mitochondrial-processing peptidase subunit β (PMPCB) suppresses EpCAM expression, disrupts Wnt/β-catenin signaling, and induces apoptosis in EpCAM^+^ Liver-CSCs via ROS accumulation (582). Moreover, anti-EpCAM antibodies including adecatumumab and catumaxomab, which have shown efficacy in other malignancies, are being explored for HCC (583).

Elevated CD13 expression in Liver-CSCs correlates with TGF-β pathway activation and EMT, enhancing stemness and suppressing ROS (584). The CD13 inhibitor ubenimex (bestatin) reduces stemness (475, 585), while NGR-LDP-AE—a fusion protein combining a CD13-targeting peptide with lidamycin—shows significant anti-tumor effects by targeting CD13^+^ Liver-CSCs and inhibiting angiogenesis (586). Other compounds like PEG-b-PLys(Ube) (587) and CU27 (588) also exhibit strong anti-tumor activity against CD13^+^ Liver-CSCs.

The co-expression of CD13 and CD90 is pivotal in HCC, where quiescent CD13^+^ and proliferating CD90^+^ cells can interconvert. This dynamic makes combinatorial inhibition highly effective, as dual targeting synergistically reduces tumor volume more than single agents (49). Direct CD90 targeting with the agent OSU-CG5 also reduces CD90^+^ populations and inhibits tumorigenesis (589). Cross-targeting strategies are also promising; CD44-directed therapies, for instance, induce apoptosis in CD90^+^ cells and hinder tumor formation (64). For CD44 itself, novel short peptides like the polyvalent directed peptide polymer (PDPP) and the P7 peptide demonstrate high-affinity binding as potential antibody alternatives (590, 591), while CD44 mAb-modified liposomes and the plant compound emodin are also effective against CD44^+^ CSCs (592, 593). Other notable targeted approaches include the hyaluronan synthesis inhibitor 4-methylumbelliferone (4Mu), which acts against CD47^+^ Liver-CSCs (594), and the mAb 1B50-1, which eliminates Liver-CSCs by binding the α2δ1 subunit (595).

In conclusion, effectively combating Liver-CSC heterogeneity requires combinatorial approaches targeting multiple markers simultaneously, offering new possibilities for personalized HCC management.

### 10.2 Targeting Liver-CSCs-associated signaling pathways

Disrupting core signaling pathways in Liver-CSCs represents a pivotal strategy to overcome therapeutic resistance in HCC (**Figure 5**). Relevant clinical trials are summarized in **Table 3**.

**Table 3:** Clinical trials targeting signaling pathways in liver-CSCs.

Targeted pathway	NCT number	Conditions	Interventions	Enrollment	Locations	Phases	Study status	Study results
Hedgehog pathway								
	NCT02151864	Hepatocellular carcinoma	LDE225	9	United States	Phase 1	Completed	No
	NCT01546519	Advanced solid malignancies (including hepatocellular carcinoma)	Vismodegib	31	United States	Phase 1	Completed	Yes
AKT pathway								
	NCT01239355	Hepatocellular carcinoma	MK2206	15	United States	Phase 2	Terminated	Yes
	NCT01425879	Hepatobiliary cancers (including hepatocellular carcinoma)	MK2206	8	United States	Phase 2	Completed	Yes
WNT pathway								
	NCT06600321	Hepatocellular carcinoma	ALN-BCAT and Pembrolizumab	158	United States	Phase 1	Recruiting	No
	NCT03645980	Hepatocellular carcinoma	DKN-01 and Sorafenib	70	Germany	Phase 1/2	Unknown	No
	NCT02069145	Hepatocellular carcinoma	OMP-54F28 and Sorafenib	10	United States	Phase 1	Completed	No
NOTCH pathway								
	NCT03422679	Advanced solid malignancies (including hepatocellular carcinoma)	CB-103	79	Multi-country	Phase 1/2	Terminated	Yes
TGF-β pathway								
	NCT02178358	Hepatocellular carcinoma	LY2157299 and Sorafenib	132	Multi-country	Phase 2	Completed	Yes
	NCT02906397	Hepatocellular carcinoma	Galunisertib and Stereotactic Body Radiotherapy (SBRT)	15	United States	Phase 1	Completed	No
	NCT02240433	Hepatocellular carcinoma	Galunisertib (LY2157299) and Sorafenib	9	Japan	Phase 1	Completed	No
NF-κB pathway								
	NCT04785287	Advanced solid malignancies (including hepatocellular carcinoma)	BMS-986218, Nivolumab, and Radiation	13	United States	Phase 1/2	Active not recruiting	No
STAT3 pathway								
	NCT01839604	Hepatocellular carcinoma	AZD9150	58	Multi-country	Phase 1	Completed	Yes
	NCT03195699	Advanced solid malignancies (including hepatocellular carcinoma)	TTI-101	60	United States	Phase 1	Active not recruiting	No

The Wnt/β-catenin pathway, activated in over 30% of HCC cases, presents a key therapeutic opportunity. FH535 directly inhibits this pathway, suppressing CD133^+^/EpCAM^+^ Liver-CSC proliferation and self-renewal (596), with demonstrated synergy alongside sorafenib through dual blockade of RAS/RAF/MAPK and Wnt/β-catenin pathways (597). Parallel strategies focus on upstream regulators: matrine derivatives WM130 and MASM suppress the AKT/GSK-3β/β-catenin axis to reduce stemness in EpCAM^+^ populations (598–600), while sophocarpine concurrently inhibits this pathway and TGF-β-induced EMT, effectively depleting Liver-CSC reservoirs in preclinical models (601).

Targeting the TGF-β pathway effectively counters Liver-CSC plasticity. The TGF-βRI inhibitor galunisertib suppresses the stemness phenotype by modulating CD44 expression, thereby impairing liver spheroid formation and invasiveness (602). Likewise, SMAD inhibitor (e.g., SB431542) promotes differentiation and chemosensitizes Liver-CSCs to chemotherapy (395), while the natural compound ovatodiolide suppresses YAP1-driven CSC phenotypes and enhances chemotherapy sensitivity (409).

The JAK/STAT pathway represents another promising target. Inhibition with TG101209 and AZ960 reduces proliferation in tumorigenic SP/CD44^+^ cells (603), while momelotinib suppresses tumor growth by targeting JAK2 and downregulating PARP1 through the IFNGR-JAK2-STAT1-PARP1 axis (604). Complementing these approaches, ZnAs@SiO_2_ nanoparticles suppress metastasis by inhibiting stemness and EMT through SHP-1-mediated blockade of JAK2/STAT3 signaling (605).

The IL-6/STAT3 pathway is a pivotal driver of HCC, making its disruption critical for targeting Liver-CSCs (392, 606–608). Clinically, the IL-6R blocker tocilizumab reduced self-renewal of CD44^+^ Liver-CSCs in a phase I trial (NCT02536469) (609). Mechanistically, this axis can be disrupted by multiple agents: S31–201 and IL-6-neutralizing antibodies directly block IL-6; madindoline A disrupts IL-6/IL-6R/gp130 complex formation; and C188–9 or curcumin inhibit STAT3 phosphorylation, preventing its oncogenic activity (608).

NOTCH signaling inhibition shows significant promise through gamma-secretase inhibitors (GSIs). L-685,458 and DAPT inhibit proliferation and stemness of EpCAM^+^ Liver-CSCs (610), while PF-03084014 reduces self-renewal and tumor growth *in vivo* and shows synergy with sorafenib (611, 612). MRK003 uniquely targets a non-canonical NOTCH pathway, significantly impairing sphere-forming capacity and depleting the Liver-CSCs pool (613).

Targeting the Hh pathway is another viable strategy. The GLI inhibitor GANT61 demonstrates significant anti-proliferative effects on CD44^+^ HCC models and reverses sorafenib resistance (614), while the natural compound berberine inhibits the PARD3-mediated Shh pathway, reducing CD133^+^ Liver-CSC self-renewal capacity (615).

In conclusion, targeting Liver-CSC signaling pathways is essential to overcome HCC therapy resistance. Developing next-generation inhibitors and understanding pathway crosstalk will be key to achieving durable tumor control.

### 10.3 Targeting the Liver-CSCs niche

Targeting the Liver-CSCs niche is crucial as the surrounding microenvironment significantly influences their stem-like traits. Notably, CAFs, TAMs, and hypoxia have a particularly significant impact. Targeting these elements could lead to substantial therapeutic advancements.

Within the HCC TME, CAFs play a crucial role in maintaining Liver-CSCs. While complete CAF depletion may risk aggressive tumor progression, as demonstrated in pancreatic models where α-SMA^+^ CAF elimination exacerbated disease (616), emerging strategies enable more selective targeting. Recent advances identify specific protumorigenic human CAF subpopulations marked by CD10 and GPR77, with neutralizing antibodies against this subset suppressing tumor growth and restoring chemotherapy sensitivity by disrupting stemness support in preclinical models (617). Alternatively, targeting HSCs—primary CAF precursors—via genetic depletion (e.g., Lrat-Cre; iDTR models) reduces tumor burden by inhibiting differentiation into both MyCAF and iCAF subsets (618).

Multiple strategies have been developed to target TAMs in HCC, focusing on three main approaches: depleting TAM populations, repolarizing their phenotype, and disrupting their protumorigenic functions (619). For instance, C-C chemokine receptor type 2 (CCR2) antagonists inhibit monocyte recruitment and differentiation into TAMs via CCL2/CCR2 axis blockade (620, 621), while colony-stimulating factor 1 receptor (CSF-1R) inhibitors such as PLX3397 repolarize immunosuppressive M2-TAMs toward antitumor M1 phenotypes (622). Additionally, adoptive cell therapy using GPC3-targeted chimeric antigen receptor macrophages (CAR-Ms) demonstrates promise through antigen-specific phagocytosis, tumor clearance, and M1 phenotype adoption in HCC models (623).

Hypoxia-directed strategies show significant clinical translation. Digoxin suppresses HIF-1α translation, inhibiting tumor growth and MDSC recruitment by disrupting HIF-1/LOX-mediated premetastatic niche formation (624, 625). Similarly, the HIF-1α-targeting agent RO7070179 (EZN-2968) yielded partial response/stable disease in 25% of HCC patients in a Phase Ib trial (626). Complementarily, HIF-2α inhibitor PT-2385 enhances sorafenib efficacy by blocking heterodimerization, suppressing tumor growth while potentially mitigating toxicity (627). Hypoxia-activated prodrugs (HAPs) like tirapazamine combined with trans-arterial embolization (TAE) achieved 84% overall response rate (ORR) in treatment-naïve unresectable HCC (628) and maintained 65% ORR in TACE-refractory patients (629), while evofosfamide plus sorafenib induced disease control in 55.6% of patients (630). Likewise, the reoxygenating compound myo-inositol trispyrophosphate (ITPP) demonstrated morphological stabilization in 52% of patients and synergistic effects with subsequent chemotherapy, achieving 70% disease control while reducing angiogenic markers in 60% of patients - correlating with improved survival outcomes (631).

Overall, targeting the Liver-CSC niche presents a promising therapeutic strategy for HCC. Future efforts should prioritize combinatorial approaches that disrupt multiple niche components to overcome therapy resistance and improve patient outcomes.

### 10.4 Other approaches to target Liver-CSCs

Beyond targeting specific markers, signaling pathways, and the niche, several other strategies have emerged to combat Liver-CSCs. These include disrupting their metabolic support, inducing differentiation, and exploiting vulnerabilities such as ferroptosis. Each approach offers unique opportunities to enhance HCC treatment efficacy.

#### 10.4.1 Targeting metabolic support

Targeting the metabolic dependencies of Liver-CSCs represents a promising therapeutic strategy for HCC. As established in this review, Liver-CSCs exhibit distinct metabolic signatures that diverge from non-CSCs populations, enabling enhanced nutrient utilization, adaptive responses to microenvironmental stress, and increased survival (632, 633). Energy disruptors—including biguanides, 2-deoxyglucose (2-DG), and aminoimidazole-4-carboxamide ribonucleoside (AICAR)—target mitochondrial function, glycolysis, and AMPK signaling to impair bioenergetic capacity (634, 635). The molecular chaperone HSP90 is critical for Liver-CSC metabolic plasticity and oncogenic processes (632, 636, 637). Notably, monoclonal antibody 11C9 targets cell-surface HSP90 on Liver-CSCs (637), while the inhibitor AUY922 (luminespib) demonstrates dose-dependent anti-proliferative effects (638). Furthermore, inhibition of GLS1 via CRISPR/Cas9-mediated knockout and pharmacologic inhibition (968/BPTES) has been shown to attenuate stemness properties by elevating ROS and suppressing the WNT/β-catenin pathway (456). Mitochondrial biogenesis further represents a therapeutic vulnerability, with salinomycin and epigallocatechin-3-gallate (EGCG) modulating organellar dynamics to alter CSC metabolic responses (633). To sum up, targeting these metabolic axes offers a rational strategy to enhance Liver-CSC eradication in HCC therapy.

#### 10.4.2 Inducing differentiation

Inducing the differentiation of Liver-CSCs into non-tumorigenic cell types is a promising strategy to deplete the self-renewing population driving HCC. Multiple approaches demonstrate efficacy, including cytokine signaling, epigenetic reprogramming, and transcriptional regulation. For instance, the cytokine OSM promotes the differentiation of quiescent EpCAM^+^ Liver-CSCs and acts synergistically with 5-FU to eliminate both CSC and non-CSC populations (639). Similarly, all-trans retinoic acid induces differentiation and inhibits malignant behaviors via retinoid signaling pathways (640, 641). A recent advance uses a small molecule cocktail (SMC) such as SB431542 (TGF-β inhibitor), CHIR99021 (GSK3β inhibitor), and BIX01294 (G9a histone methyltransferase inhibitor), to epigenetically reprogram drug-resistant cells toward hepatocyte-like differentiation, causing tumor regression via AKT/mTOR/HIF1α modulation (642). Transcription factor-directed strategies are also effective; HNF4α activates hepatocyte differentiation programs to suppress stemness in CD90^+^/CD133^+^ Liver-CSCs (643), while bone morphogenetic protein 4 (BMP4) induces differentiation of CD133^+^ Liver-CSCs to reduce their tumorigenicity (644). Collectively, these differentiation-inducing agents comprise a targeted therapeutic approach to undermine Liver-CSC maintenance and mitigate HCC malignancy.

#### 10.4.3 Inducing ferroptosis

Liver-CSCs exhibit a unique dependency on iron, often referred to as “iron addiction,” which is critical for their survival and proliferation (645, 646). This elevated iron content supports essential cellular functions, such as energy production and DNA synthesis, but also renders Liver-CSCs susceptible to ferroptosis, an iron-dependent form of regulated cell death characterized by the accumulation of ROS and lipid peroxidation (646). Ferroptosis is primarily triggered by lipid peroxidation and is tightly regulated by proteins such as solute carrier family 7, member 11 (SLC7A11), a component of the cystine-glutamate antiporter system (647). The stem cell marker CD44 plays a pivotal role in this context. CD44 mediates the endocytosis of iron-bound hyaluronates, contributing to the elevated iron levels within Liver-CSCs (648). Additionally, CD44 stabilizes SLC7A11, supporting the synthesis of glutathione, a potent antioxidant that neutralizes ROS and prevents lipid peroxidation, thereby protecting Liver-CSCs from ferroptosis (647). This dependency can be therapeutically exploited. Artesunate induces ferroptosis by disrupting the labile iron pool and induces ER-derived ROS-mediated cell death (649, 650). Similarly, sorafenib, sulfasalazine, and ras-selective lethal small molecule 3 (RSL3) trigger ferroptosis by inhibiting the cystine/glutamate antiporter or directly targeting peroxidase 4 (GPX4) (651, 652). These findings underscore the potential of inducing ferroptosis as a strategy to target Liver-CSCs, though clinical validation is required.

## 11 Future perspectives

The central role of Liver-CSCs in driving the therapeutic resistance, recurrence, and metastasis of HCC is now undeniable. However, translating this biological understanding into clinical success requires navigating a complex landscape of interconnected challenges. A strategic path forward must therefore address several critical fronts, which include—but are not limited to—resolving fundamental questions of cellular identity, pioneering novel targeting paradigms, and revolutionizing translational models and clinical trial design.

### 11.1 Decoding cellular origins to unlock therapeutic vulnerabilities

A fundamental, unresolved question is whether Liver-CSCs from different cellular origins—such as hepatic progenitors, mature hepatocytes, cell fusion, or via dedifferentiation of non-CSCs—possess unique functional properties and therapeutic susceptibilities. Future research must move beyond mapping these pathways and instead focus on functionally interrogating how each origin dictates a distinct “therapeutic identity.” Addressing this question is critical; as it could reveal whether a single universal therapy is viable or if a more personalized arsenal of origin-specific regimens might be required for effective Liver-CSC eradication.

### 11.2 Moving beyond surface markers: targeting the core regulatory state and plasticity

Building on the need to understand cellular identity, the field is currently constrained by the non-specificity of surface markers (653, 654) and the profound cellular plasticity of Liver-CSCs (558–560), which allows them to dynamically switch states and evade static targeting. A transformative breakthrough lies in leveraging multi-omics integration—simultaneously profiling the epigenome, transcriptome, and proteome at single-cell resolution—to define a dynamic “epigenetic barcode” of the core Liver-CSC state. Furthermore, this approach should be expanded through metabolomic and lipidomic profiling to systematically uncover their unique metabolic dependencies. Unlike differentiated cells, Liver-CSCs exhibit metabolic plasticity, shifting between glycolysis, oxidative phosphorylation, and fatty acid oxidation to survive therapy. This integrated multi-omics strategy could pinpoint specific vulnerabilities, such as dependencies on key metabolic enzymes or lipid storage mechanisms, potentially revealing novel targets to disrupt their self-renewal and eradicate the root of therapeutic resistance (655–657).

Importantly, this multi-omics approach is particularly valuable for identifying superior therapeutic targets like cancer-testis antigens (CTAs) including melanoma-associated antigen A9 (MAGE-A9) and New York esophageal squamous cell carcinoma 1 (NY-ESO-1). Their value is twofold: first, they exhibit near-absolute tumor specificity, being expressed primarily in malignant cells (including Liver-CSCs) and immune-privileged germ cells, but not in normal adult tissues (658–661). This creates a wide therapeutic window, minimizing on-target, off-tumor toxicity. Second, they are functionally linked to the core stemness circuitry, playing direct roles in maintaining self-renewal and tumorigenicity (660–662). This makes them ideal targets for sophisticated immunotherapies, such as TCR-engineered T cells or cancer vaccines.

Beyond identifying new targets, the therapeutic paradigm must shift from targeting static markers to disrupting the drivers of cellular plasticity. This involves developing strategies aimed at collapsing the transient “stemness window”—the high-plasticity state that confers maximal tumor-initiating potential (561–563). Promising agents, such as SPINK1-neutralizing antibodies, exemplify this approach by preventing the phenotypic switching that underlies adaptive chemoresistance and relapse (499).

### 11.3 Bridging the translational gap with advanced models and clinical innovation

To effectively translate these biological insights into clinical benefits, we must address the high failure rate of clinical trials for Liver-CSC-targeted therapies, which primarily stems from using models that ignore fundamental biological realities. First, the absence of physiological hypoxia in conventional preclinical models is a critical flaw (663, 664). Since hypoxia actively drives the Liver-CSC phenotype, upregulating stemness markers, promoting quiescence, and enhancing therapy resistance. Therefore, next-generation models must enforce controlled, physiological hypoxia cycles to test therapies against the most therapeutically relevant Liver-CSC subpopulations. Second, moving beyond “immune-blind” systems is crucial. Liver-CSCs shielded by patient-derived CAFs and TAMs exhibit completely different drug responses. These advanced, hypoxic, immune-competent PDOs could function as preclinical “avatar” trials to identify drug combinations that penetrate the niche (665).

However, even the most promising therapy will fail in the clinic if the trial design cannot accurately detect its effect. The fundamental challenge lies in the mismatch between conventional endpoints—response evaluation criteria in solid tumors (RECIST criteria), OS, and PFS—and the biological reality of Liver-CSCs as a critical but variable tumor subpopulation. When a therapy demonstrates potent efficacy in a biomarker-defined patient group, this signal becomes diluted to the point of statistical insignificance when averaged across an unselected cohort in traditional trial designs. This necessitates the integration of Liver-CSC-specific biomarkers, particularly measurable indicators like reduction in circulating Liver-CSCs, which serve the dual purpose of providing biological proof-of-concept and enabling precise identification of responding patients. Equipped with these diagnostic tools, the field can advance toward a paradigm of preemptive therapy—deploying plasticity-disrupting agents before standard treatments such as surgery or TACE to prevent therapy-induced expansion of the resistant cellular pool. Ultimately, the success of this strategy depends on implementing biomarker-driven adaptive trials that utilize liquid biopsy monitoring in real-time, establishing a clinical framework capable of matching the dynamic adaptability of the cancer it seeks to eliminate.

In conclusion, the path to overcoming therapeutic resistance in HCC lies not in a single breakthrough, but in a coordinated, multi-pronged campaign. The interconnected strategies of deciphering cellular origins, redefining targeting through multi-omics and plasticity disruption, and bridging the translational gap with advanced models and innovative trials are mutually reinforcing. By simultaneously attacking the problem on these fronts—understanding the ‘seed,’ disrupting its core identity, and revolutionizing the ‘soil’ in which it is tested and treated—the next decade of research holds the potential to transform our profound biological insights into lasting clinical remission for HCC patients.

## 12 Conclusion

HCC remains a major global health burden, characterized by high recurrence and therapy resistance that highlight the failure of conventional treatments targeting the bulk tumor. This review consolidates compelling evidence identifying Liver-CSCs as the central drivers of HCC aggression and treatment failure. These cells represent a dynamic, heterogeneous population defined by their core capabilities: self-renewal, differentiation, and profound plasticity.

Liver-CSC persistence is orchestrated within a specialized TME, where cellular components and non-cellular factors create a protective niche that sustains stemness and confers therapy resistance. This resilience is further enabled by an intricate network of dysregulated signaling pathways, epigenetic reprogramming, and metabolic adaptations, which together promote dormancy, enhance DNA repair, drive immune evasion, and facilitate drug efflux.

This multifaceted understanding leads to an inescapable conclusion: durable control and cure of HCC require therapeutic strategies that co-target the bulk tumor and the resilient Liver-CSC reservoir. The future of HCC management lies in rational combination therapies—integrating targeted agents, immunotherapies, and niche-disrupting compounds—guided by robust biomarker-based patient stratification. While translational challenges persist, the continued elucidation of Liver-CSC biology offers a clear and promising roadmap for overcoming therapeutic resistance and improving outcomes for patients with this devastating disease.
